# Primer, Pipelines, Parameters: Issues in 16S rRNA Gene Sequencing

**DOI:** 10.1128/mSphere.01202-20

**Published:** 2021-02-24

**Authors:** Isabel Abellan-Schneyder, Monica S. Matchado, Sandra Reitmeier, Alina Sommer, Zeno Sewald, Jan Baumbach, Markus List, Klaus Neuhaus

**Affiliations:** a Core Facility Microbiome, ZIEL—Institute for Food & Health, Technische Universität München, Freising, Germany; b Chair of Experimental Bioinformatics, TUM School of Life Sciences Weihenstephan, Technische Universität München, Freising, Germany; c Computational Biomedicine Lab, Department of Mathematics and Computer Science, University of Southern Denmark, Odense, Denmark; d Chair of Computational Systems Biology, University of Hamburg, Hamburg, Germany; U.S. Department of Energy Joint Genome Institute

**Keywords:** 16S rRNA gene sequencing, amplicon sequencing, variable regions, clustering, bioinformatic settings, microbiome, databases, mock communities

## Abstract

Short-amplicon 16S rRNA gene sequencing is currently the method of choice for studies investigating microbiomes. However, comparative studies on differences in procedures are scarce. We sequenced human stool samples and mock communities with increasing complexity using a variety of commonly used protocols. Short amplicons targeting different variable regions (V-regions) or ranges thereof (V1-V2, V1-V3, V3-V4, V4, V4-V5, V6-V8, and V7-V9) were investigated for differences in the composition outcome due to primer choices. Next, the influence of clustering (operational taxonomic units [OTUs], zero-radius OTUs [zOTUs], and amplicon sequence variants [ASVs]), different databases (GreenGenes, the Ribosomal Database Project, Silva, the genomic-based 16S rRNA Database, and The All-Species Living Tree), and bioinformatic settings on taxonomic assignment were also investigated. We present a systematic comparison across all typically used V-regions using well-established primers. While it is known that the primer choice has a significant influence on the resulting microbial composition, we show that microbial profiles generated using different primer pairs need independent validation of performance. Further, comparing data sets across V-regions using different databases might be misleading due to differences in nomenclature (e.g., *Enterorhabdus* versus *Adlercreutzia*) and varying precisions in classification down to genus level. Overall, specific but important taxa are not picked up by certain primer pairs (e.g., *Bacteroidetes* is missed using primers 515F-944R) or due to the database used (e.g., *Acetatifactor* in GreenGenes and the genomic-based 16S rRNA Database). We found that appropriate truncation of amplicons is essential and different truncated-length combinations should be tested for each study. Finally, specific mock communities of sufficient and adequate complexity are highly recommended.

**IMPORTANCE** In 16S rRNA gene sequencing, certain bacterial genera were found to be underrepresented or even missing in taxonomic profiles when using unsuitable primer combinations, outdated reference databases, or inadequate pipeline settings. Concerning the last, quality thresholds as well as bioinformatic settings (i.e., clustering approach, analysis pipeline, and specific adjustments such as truncation) are responsible for a number of observed differences between studies. Conclusions drawn by comparing one data set to another (e.g., between publications) appear to be problematic and require independent cross-validation using matching V-regions and uniform data processing. Therefore, we highlight the importance of a thought-out study design including sufficiently complex mock standards and appropriate V-region choice for the sample of interest. The use of processing pipelines and parameters must be tested beforehand.

## INTRODUCTION

The human gut microbiome is a complex environment hosting a large number of different bacteria. A cost-effective method to determine the bacterial composition of, e.g., human fecal samples is to sequence amplicons targeting the 16S rRNA gene. Microbial compositions of diverse environments, which are influenced by different factors or conditions (e.g., sampling time point, targeted rRNA region, response to health or disease, sequencing strategy, machinery, depth, and read lengths), were also studied with this method (1–7).

The 16S rRNA gene spans about 1,500 bp and is structured in highly conserved regions interspersed with nine variable regions (V-regions), V1 to V9 (8, 9). The conserved regions can be used for primer binding and thus allow for capturing a greater number of different bacterial taxa, sometimes including or not including archaea, while the variable regions permit the discrimination of these taxa within different microbial environments (10). However, differences between the conserved regions and, therefore, differences in primer annealing result in an unequal amplification of bacteria present in a sample (11). Depending on the particular V-region that was targeted, differences in the sequencing results and taxonomic outcome occurred, which led to misinterpretation (12, 13). Further, not every variable region has the same sensitivity, i.e., allowing separation of closely related taxa (14). Concerning archaea, the applicability of certain primer pairs has been covered well in previous studies (12, 15, 16).

Second-generation sequencers, e.g., Illumina’s MiSeq, enable sequencing of amplicons up to 600 bp with high accuracy. This length allows targeting about one to three adjacent variable regions of the 16S rRNA gene using “universal” primers for the conserved regions. In a subsequent PCR, sequencing adapters are added to the amplicons (17). After a cleanup step, the amplicon libraries are sequenced. The resulting reads are used to analyze similarities and differences between samples with different microbial compositions (e.g., alpha- and beta-diversity) (18). In contrast, full-length 16S rRNA gene sequencing is possible by using third-generation sequencers, for instance, Oxford Nanopore MinION (19) and the PacBIOs Sequel (20), which were introduced in 2009 and 2008, respectively. The greatest advantage is the long read length (up to 10,000 bp) and sequencing on a single-molecule level in a short time. These long reads enable an improved identification of bacterial taxa, as shown in several recent studies (21–27). Nevertheless, significant drawbacks include the relatively high error rate (up to 15% per sequence) (28, 29), limited applicability in high-throughput studies, higher general costs, and even less standardization of protocols and analysis pipelines. However, despite the widespread use of 16S rRNA gene sequencing, there is a need to better understand the differences between the targeted region and the data analysis pipeline chosen in amplicon sequencing of the 16S rRNA genes.

For short-amplicon sequencing, a literature survey showed that the regions V1-V2/V3 (30, 31), V3-V4/V5 (32–34), and V4 (35, 36) are most commonly used. However, the taxonomic classification differs considerably when targeting different variable regions (37), affecting attempts to perform cross-study comparison and leading to further biases in compositional analysis, where short-amplicon primers are not as universal as desired (11, 38). Since the taxonomic resolution seems to differ for some phyla for different variable regions (39), closely related bacterial species and genera might be indistinguishable (40). Moreover, the choice of bioinformatic processing pipelines and analysis tools is known to influence the results (41–44). Different 16S rRNA gene-specific taxonomic classification methods, such as Mothur (45), Qiime (46), Qiime2 (47), DADA2 (48), and others, were developed. During data processing, sequences are clustered into operational taxonomic units (OTUs) at a threshold of 97% sequence similarity. Sequence representatives, i.e., sequences with the least mismatches to other sequences in a cluster, are used for taxonomic assignment. Amplicon sequence variants (ASVs) or zero-radius OTUs (zOTUs) have been suggested as alternatives to OTUs (48, 49), as they correct for sequencing errors by different denoising approaches. In contrast to OTUs, these clusters are supposed to contain reads originating only from the same bacterial species, enabling a cross-study comparison (49, 50). In any case, after clustering, sequences are classified for taxonomic assignment using databases of known 16S rRNA gene sequences, e.g., GreenGenes (GG) (51), the Ribosomal Database Project (RDP) (52), Silva (53), the genomic-based 16S rRNA Database (GRD) (54), or The All-Species Living Tree (LTP) (55). Not only different pipelines and reference databases but also settings of a given pipeline influence the results and are an often-overlooked bias in microbiome studies (42, 56–58). Nevertheless, some biases occurring in 16S rRNA gene amplicon sequencing have already been addressed in the past. Well-studied biasing factors, for instance, include sampling and storage procedures (59–63), DNA extraction methods (64–68), choice of variable region and primers (12, 36, 69–72), library preparation and sequencing strategies (73–76), and sequence data processing, including denoising, taxonomic classification, and the use of distinct bioinformatic tools (42, 56–58). Further, the use of negative controls and mock communities as internal standards to detect contamination or aberrancies in the sequencing results was proposed (77–79).

In this study, we joined several of these separate issues to raise awareness that the combination of primer sequence choice, clustering methods, reference database, and analysis parameters must be considered thoroughly to avoid increased bias. Thus, we created a large benchmark data set of 16S rRNA gene amplicon sequences, targeting different V-regions of the 16S rRNA gene, and systematically tested different software tools with different sets of parameters for the analysis. We sequenced three mock communities of increasing complexity with known composition, along with complex human fecal samples for comparison.

## RESULTS

**FIG 1:**
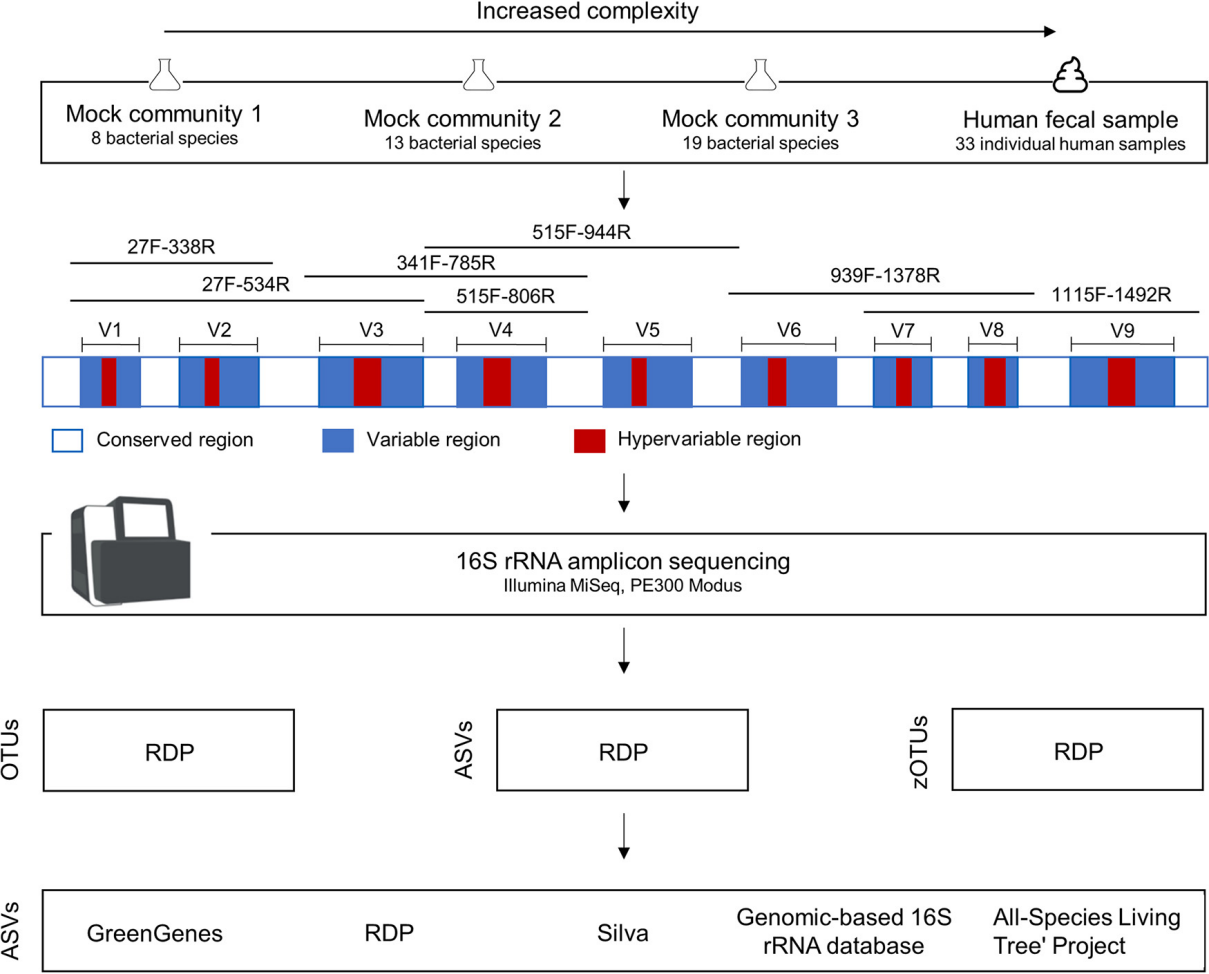
Overview of the analysis strategies used in this study. DNAs from different sample types with increasing complexity (i.e., 3 mock communities and 33 human stool samples) were extracted. Amplicons were generated using different primer pairs targeting different V-regions and sequenced on an Illumina MiSeq. Afterwards, the impacts of different clustering approaches and reference databases on the microbial profiles were investigated.

**FIG 2:**
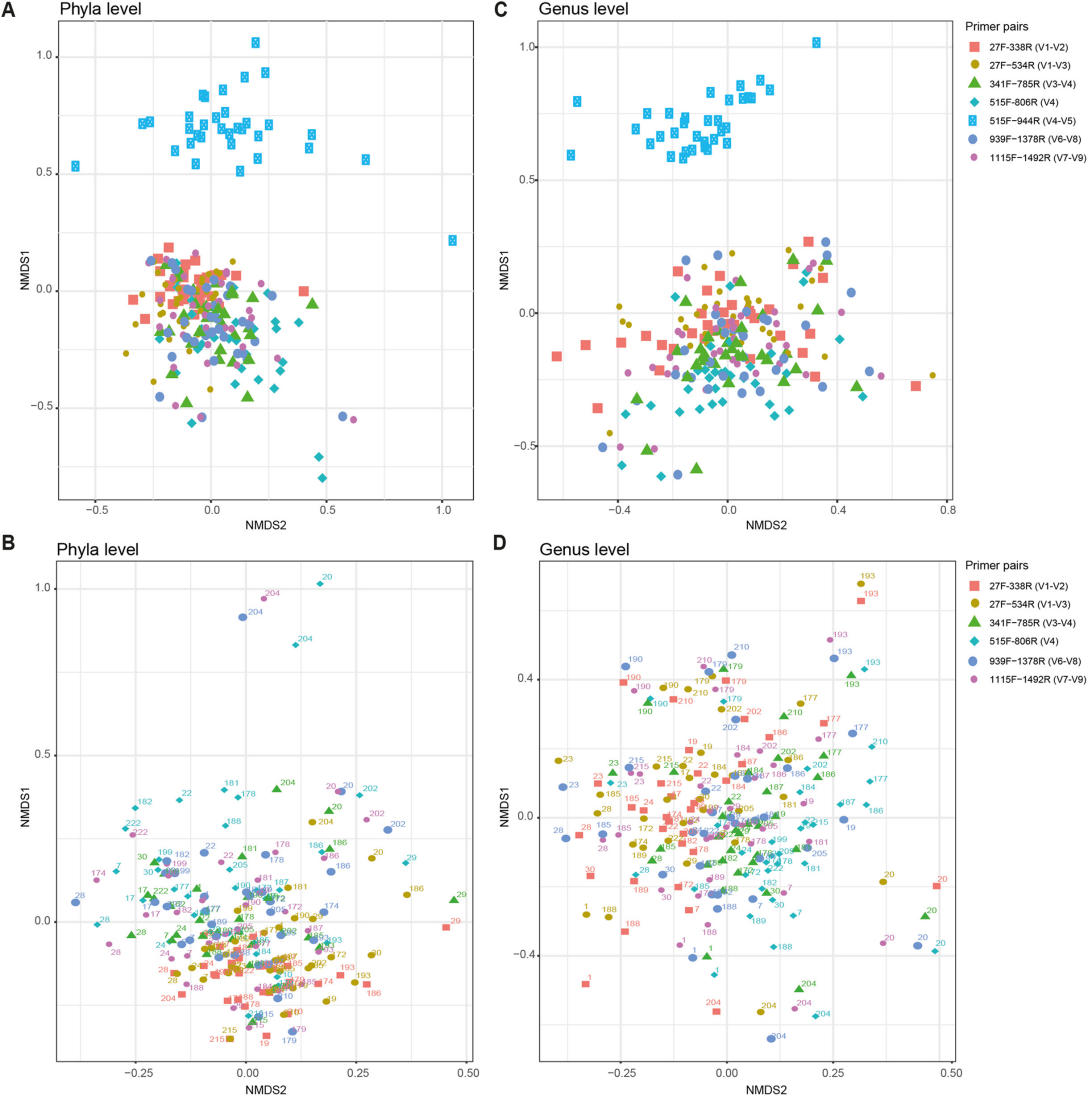
NMDS plots for the microbiome composition of human samples. Sample similarity is shown at phylum level (A and B) and at genus level (C and D). Different primer pairs are indicated to the right for all panels. Top panels (A and C) include processing the V4-V5 region, while for the bottom panels (B and D) this region has been omitted since results using 515F-944R primers (blue squares in panels A and C) fall separately from all other clusters. Labeling of the samples in the bottom panels (B and D) is based on donor number.

We systematically assessed the global influence of multiple parameters in mock communities of known composition and in human samples (Fig. 1). First, the choice of primers targeting different variable regions of the 16S rRNA gene was evaluated. We show that primer choice influences the taxonomic composition, visible in a multidimensional scaling (MDS) plot of samples originating from the same donor (Fig. 2). Second, we investigated how, and in what magnitude, the use of different clustering approaches and taxonomy assignment methods influences the results for the classification of bacterial taxonomies.

### Primer choice influences the estimated microbial composition.

**FIG 3:**
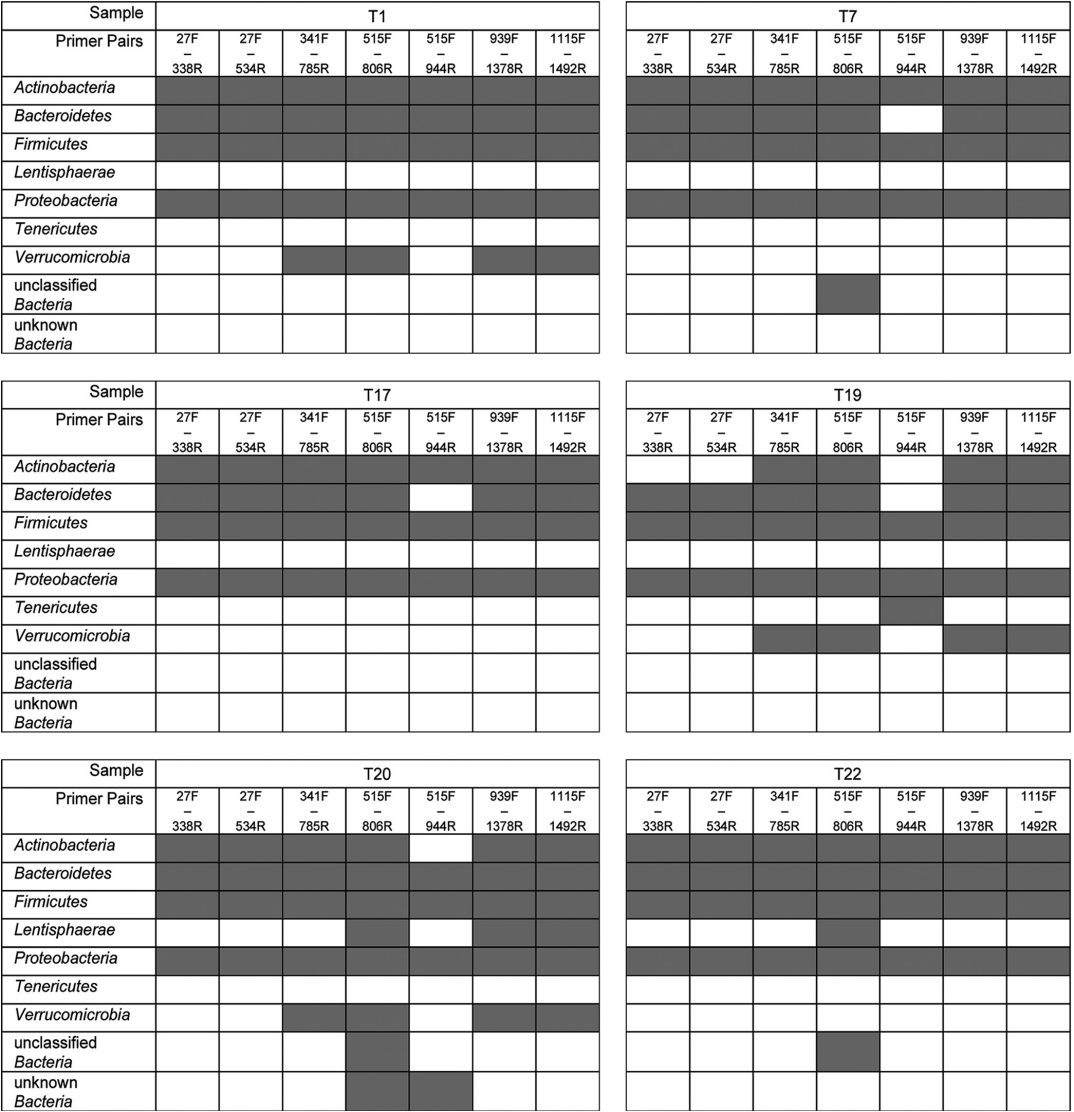
Presence-and-absence map of human samples on phylum level for different V-regions. Gray represents present taxa, and white represents absent taxa. Primers and their V-region spanning are given in Table 1.

A set of different 16S rRNA gene sequencing primer pairs covering one, two, or three of the variable regions V1 to V9 is commonly used for the analysis of microbial compositions. Depending on the input material (e.g., human gut samples, water analysis, sludge, food research, etc.), different primer pairs are used. In this study, we investigated seven different primer pairs, 27F-338R (V1-V2), 27F-534R (V1-V3), 341F-785R (V3-V4), 515F-806R (V4), 515F-944R (V4-V5), 939F-1378R (V6-V8), and 1115F-1492R (V7-V9), for the analysis of human gut samples and mock communities (Fig. 1 and Table 1). The use of different primer pairs led to primer-specific and not mainly donor-specific clustering of human stool samples (Fig. 2). These differences varied according to the analyzed taxonomic level. Differences were found to be less pronounced at higher taxonomic levels, e.g., phylum level compared to genus level (Fig. 2A and C). When analyzing samples from the same human donor but sequenced using different primer pairs, some taxa are unique for certain primer pairs. For instance, when analyzing human sample 1 (Fig. 3), *Verrucomicrobia* was detected only when using 341F-785R (V3-V4), 515F-806R (V4), 939F-1378R (V6-V8), and 1115F-1492R (V7-V9) primers and not 27F-338R (V1-V2), 27F-534R (V1-V3), or 515F-944R (V4-V5). Comparisons of samples derived from the same human donor but sequenced using different primer pairs become even more difficult at the genus level (see Fig. S2 in the supplemental material). This was mainly due to differences in the prevalence of genera when using different V-regions. A large number of reads were not classified down to genus level in either one or several V-regions and were thus considered “unknown.” Importantly, the 515F-944R (V4-V5) primer pair seemed to produce results with only a few overlaps with other primer pairs (Fig. 2) and displayed a low abundance of *Bacteroidetes* (Fig. 3; Fig. S2). We analyzed whether this was due to a much lower theoretical coverage of known bacterial species. Therefore, all primers were evaluated *in silico* for their theoretical coverage on all bacterial genera using the Silva database. While the theoretical coverage for 515F-944R (V4-V5) primers was lower than for the primer pairs 27F-338R (V1-V2), 27F-534R (V1-V3), 341F-785R (V3-V4), and 515F-806R (V4), we found the theoretical coverage for primer pairs 939F-1378R (V6-V8) and 1115F-1492R (V7-V9) to be even lower (Table S2). Thus, we believe that the low coverage of *Bacteroidetes* is the main reason for primer pair 515F-944R (V4-V5) to form an outlier.

**TABLE 1:** V-region-specific forward and reverse primers and annealing temperature for 1st step PCR

V-region	Forward primer	Reverse primer	Forward sequence (5′–3′)	Reverse sequence (5′–3′)	Specificity	Annealing temp (°C)	Reference
V1-V2	27F	338R	AGA GTT TGA TYM TGG CTC AG	GCT GCC TCC CGT AGG AGT	Universal^*a*^	57	Salter et al. (115)
V1-V3	27F	534R	AGA GTT TGA TYM TGG CTC AG	ATT ACC GCG GCT GCT GG	Universal	57	Walker et al. (84)
V3-V4	341F	785R	CCT ACG GGN GGC WGC AG	GAC TAC HVG GGT ATC TAA TCC	Universal	55	Klindworth et al. (70)
V4	515F	806R	GTG CCA GCM GCC GCG GTA A	GGA CTA CHV GGG TWT CTA AT	Universal	53	Caporaso et al. (116)
V4-V5	515F	944R	GTG CCA GCM GCC GCG GTA A	GAA TTA AAC CAC ATG CTC	Bacterial	53	Fuks et al. (117)
V6-V8	939F	1378R	GAA TTG ACG GGG GCC CGC ACA AG	CGG TGT GTA CAA GGC CCG GGA ACG	Bacterial	58	Lebuhn et al. (118)
V7-V9	1115F	1492R	CAA CGA GCG CAA CCC T	TAC GGY TAC CTT GTT ACG ACT T	Bacterial	51	Turner et al. (119)

a Universal, binds to archaea and bacteria.

10.1128/mSphere.01202-20.2 FIG S2 Human samples at genus level, using GG (A), RDP (B), Silva (C), GRD (D), and LTP (E) as reference databases. The primer pairs span the following V-regions: 27F-338R, V1-V2; 27F-534R, V1-V3; 341F-785R, V3-V4; 515F-806R, V4; 515F-944R, V4-V5; 939F-1378R, V6-V8; and 1115F-1492R, V7-V9. Download FIG S2, PDF file, 0.7 MB. Copyright © 2021 Abellan-Schneyder et al. 2021 Abellan-Schneyder et al. https://creativecommons.org/licenses/by/4.0/ This content is distributed under the terms of the Creative Commons Attribution 4.0 International license.

10.1128/mSphere.01202-20.5 TABLE S2 *In silico* evaluation for used primers. Download Table S2, PDF file, 0.02 MB. Copyright © 2021 Abellan-Schneyder et al. 2021 Abellan-Schneyder et al. https://creativecommons.org/licenses/by/4.0/ This content is distributed under the terms of the Creative Commons Attribution 4.0 International license.

### Clustering approaches have minor influence on taxonomic profiles.

**FIG 4:**
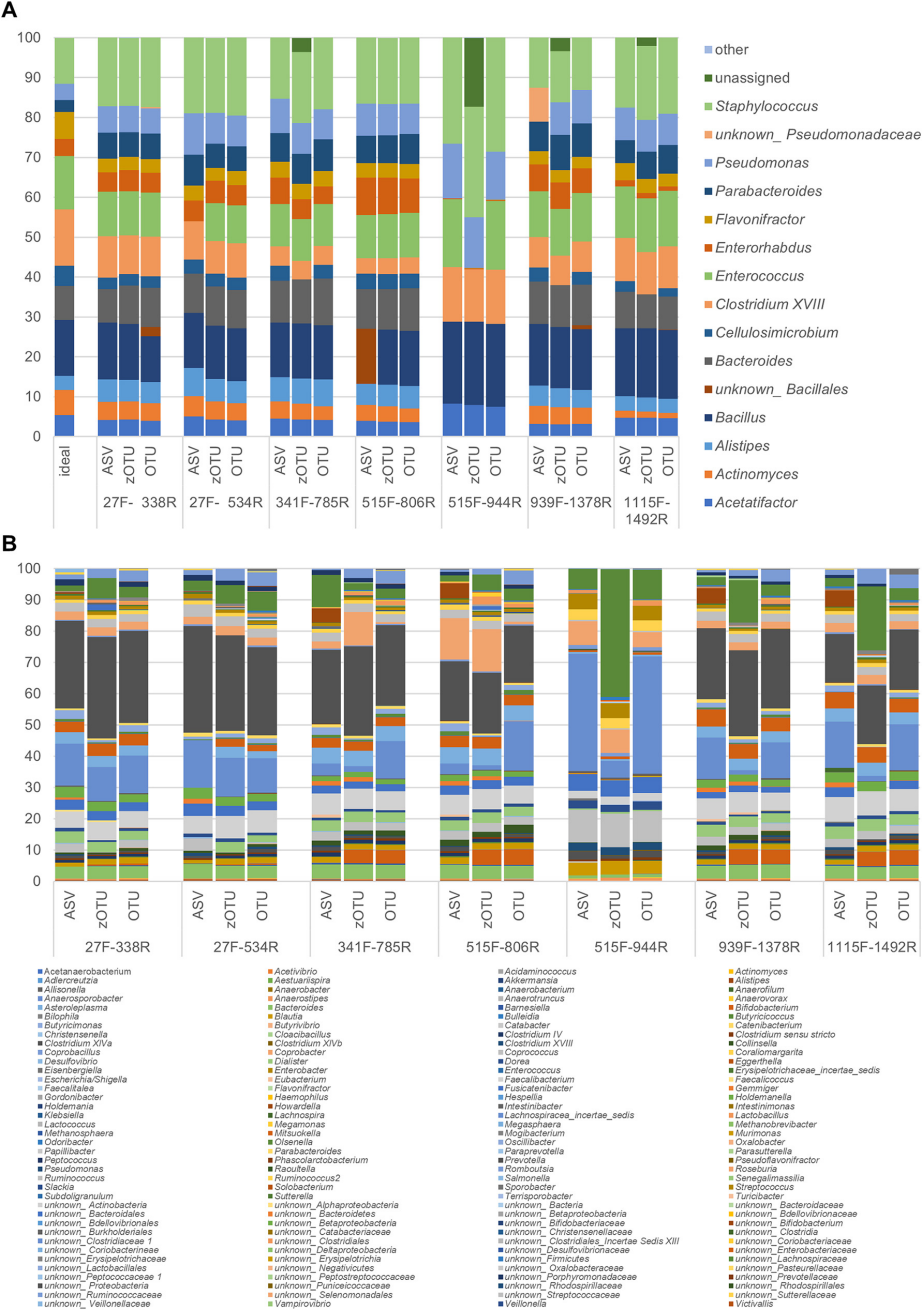
Comparison of the influence of the clustering method on taxonomic designation for the ZIEL-I mock community (A) and an example of a representative human sample T1 (B). The genus-level composition is shown according to ASVs, zOTUs, and OTUs as indicated. “Other” represents taxa not matching the composition of the mock community, while “unassigned” represents reads that could not be assigned to any taxonomic classification (RDP was used as a reference database). Primers and their V-region spanning are given in Table 1.

In addition to the 97%-identity OTU approach, ASV clustering gained a lot of attention in the latest studies (43). Due to its improved resolution and thus better comparability of results between different studies, it is nowadays a popular and often favored method. In this study, we tested whether different clustering approaches have an influence on the assigned taxonomic profiles for the ZIEL-I mock community. Thus, we compared ASVs, zOTUs, and OTUs. Overall, the clustering methodology seemed to have only a minor effect on the assigned taxonomic composition compared to the effect of primer choice (Fig. 4A). Again, the 515F-944R (V4-V5) primer pair showed profiles distinct from those found for all other primer pairs used, no matter which clustering was used. Differences observed for each clustering approach were mainly due to identification problems at the genus level. When using the ASV approach for clustering the data, *Bacillus* could not be classified down to genus level. In contrast, this was possible when using zOTU and OTU approaches. Similarly, *Enterococcus* was not assigned correctly by the 27F-534R (V1-V3) primer pair using the ASV approach. Overall, we found that ASVs performed best for most of the other genera, as differences between theoretical values and expected amounts of the distinct taxa were the smallest here (Table S3). The additional analysis of a human sample subset resulted in results comparable to those for the ZIEL-I mock community (example of one representative sample is shown in Fig. 4B). Differences in taxonomic profiles are more dependent on primer pairs used than on clustering approach. Smaller variations occurred mostly due to problems assigning genera; e.g., identification of members of the *Lachnospiraceae* family on the genus level is not possible for zOTUs when using primer pairs 515F-944R (V4-V5) and 1115F-1492R (V7-V9). Still, neither OTU nor zOTU clustering caused a larger bias, and thus, the influence of clustering is limited.

10.1128/mSphere.01202-20.6 TABLE S3 Overview of numbers and percentages of retained reads after each processing step while generating ASVs. Download Table S3, PDF file, 0.06 MB. Copyright © 2021 Abellan-Schneyder et al. 2021 Abellan-Schneyder et al. https://creativecommons.org/licenses/by/4.0/ This content is distributed under the terms of the Creative Commons Attribution 4.0 International license.

### Sample taxonomies are influenced by reference databases.

Ideally, the 16S rRNA gene sequences should reflect the organism the sequence came from. However, this depends not only on the primer pairs used or how sequence data were extracted from the raw data but also on the quality of the reference database and thus the taxonomic classification. We systematically tested five different databases commonly used: GG, RDP, Silva, GRD, and LTP.

**FIG 5:**
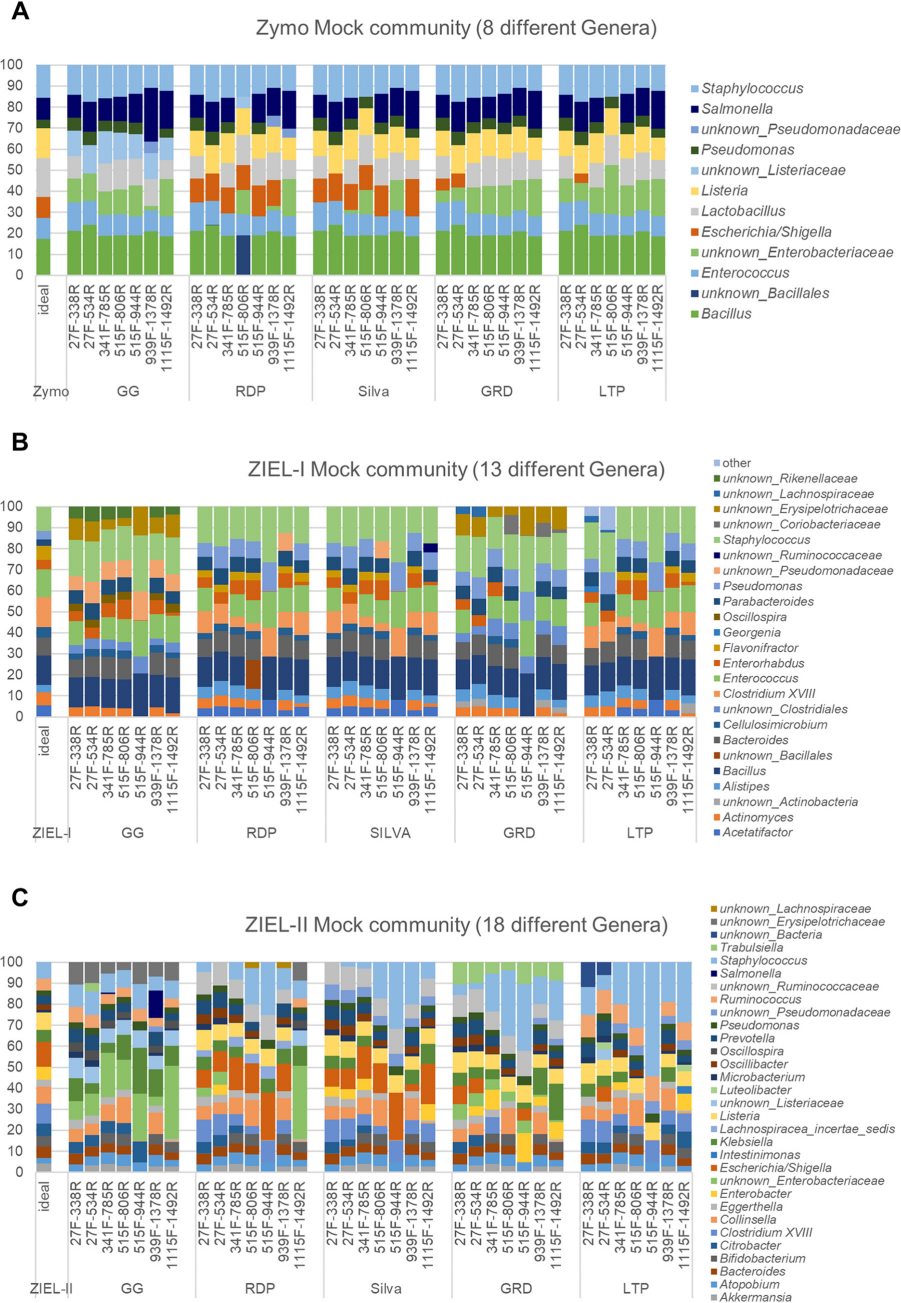
Comparison of mock communities sequenced over different V-regions, processed using different databases as references (GG, GreenGenes; RDP, Ribosomal Database Project; GRD, the genomic-based 16S rRNA database; LTP, The All-Species Living Tree Project) at genus level. Primers and their V-region spanning are given in Table 1.

When analyzing the Zymo mock community, which includes only eight different bacteria, we observed just a few minor differences in the assigned taxonomy for different V-regions used. Further, differences were relatively minor using different reference databases in the analysis (Fig. 5A). Using RDP for primer pair 515F-806R (V4), *Bacillus* could not be classified at the genus level but was at least assigned to *Bacillales* at the family level. The classification of *Escherichia/Shigella* was most accurate when using Silva or RDP as a reference database; thus, it displayed the lowest deviation from the ideal composition of the mock community. GG could not identify *Escherichia/Shigella* and *Listeria* at the genus level and showed poor results. When using the Zymo mock community, GG might be dismissed as an inferior database, but all other parameters seemed to have no major impact. However, as a mock community of only eight bacterial species provides only limited insights, we used two further, more complex mock communities.

The ZIEL-I mock community consists of 13 species in 13 genera (Fig. 5B) and uses bacteria, which would be expected in the gut. Analyzing this, GG performed worst again. No genus-level classification for *Acetatifactor*, *Bacillus*, *Clostridium*, and *Pseudomonas* was possible using GG as a reference. GRD classified neither *Bacillus* nor *Pseudomonas* down to genus level. The other databases worked reasonably well but with some differences between V-regions. As before, 515F-944R (V4-V5) data performed worst. Only 4 to 8 taxa were classified at genus level, whereas between 9 and 13 taxa (Table 2) were identified when analyzing the data generated by using the primer pair 341F-785R (V3-V4). *Actinomyces*, *Alistipes*, *Bacteroides*, *Cellulosimicrobium*, *Parabacteroides*, and *Flavonifractor* were not detected with the primer pair 515F-944R (V4-V5) at the genus level irrespective of the reference database used.

**TABLE 2:**
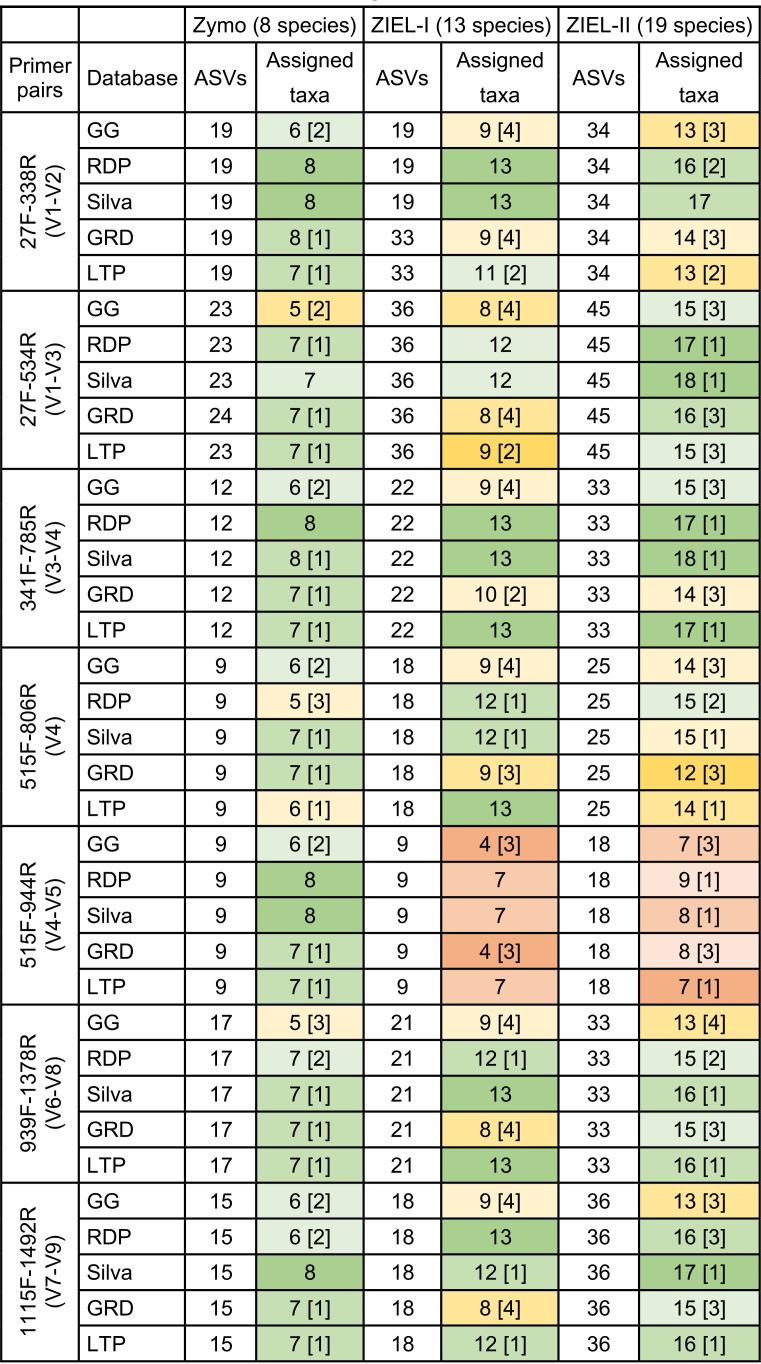
Number of ASVs and number of assigned taxa^*a*^

a Assigned taxa are at the genus level; brackets indicate that taxa are unknown at the genus level. The Zymo, ZIEL-I, and ZIEL-II mock communities contain 8, 13, and 19 bacterial species, respectively (for ZIEL-II, 18 at genus level, when *Escherichia/Shigella* fall into one cluster). Shading in green indicates good identification (the darker the better), while yellow and darker shading indicates inferior outcomes.

The ZIEL-II mock community increased the complexity of the comparison by including 19 bacteria in 18 genera. Furthermore, we purposely included species which showed difficulties in past experiments (data not shown). Again, the 515F-944R (V4-V5) primer pair showed inadequate performance irrespective of the database. Using the Silva database, 14 to 18 taxa were classified at genus level for primer pair 341F-785R (V3-V4), whereas only 7 to 9 taxa were found for data corresponding to primer pair 515F-944R (V4-V5) at genus level (Table 2). *Akkermansia* could not be identified using the 27F-338R (V1-V2) primers (Fig. 5C). *Microbacterium* was underrepresented when using the 341F-785R (V3-V4) primers. *Enterobacter* and *Ruminococcus* were best classified by Silva. Generally, most accurate taxonomic classifications were possible when using Silva or RDP as the reference database. Silva even had the smallest amount of unknown genus-level identifications, followed by RDP, LTP, GRD, and GG.

### Specific pipeline settings have minor influences on taxonomic classification.

**FIG 6:**
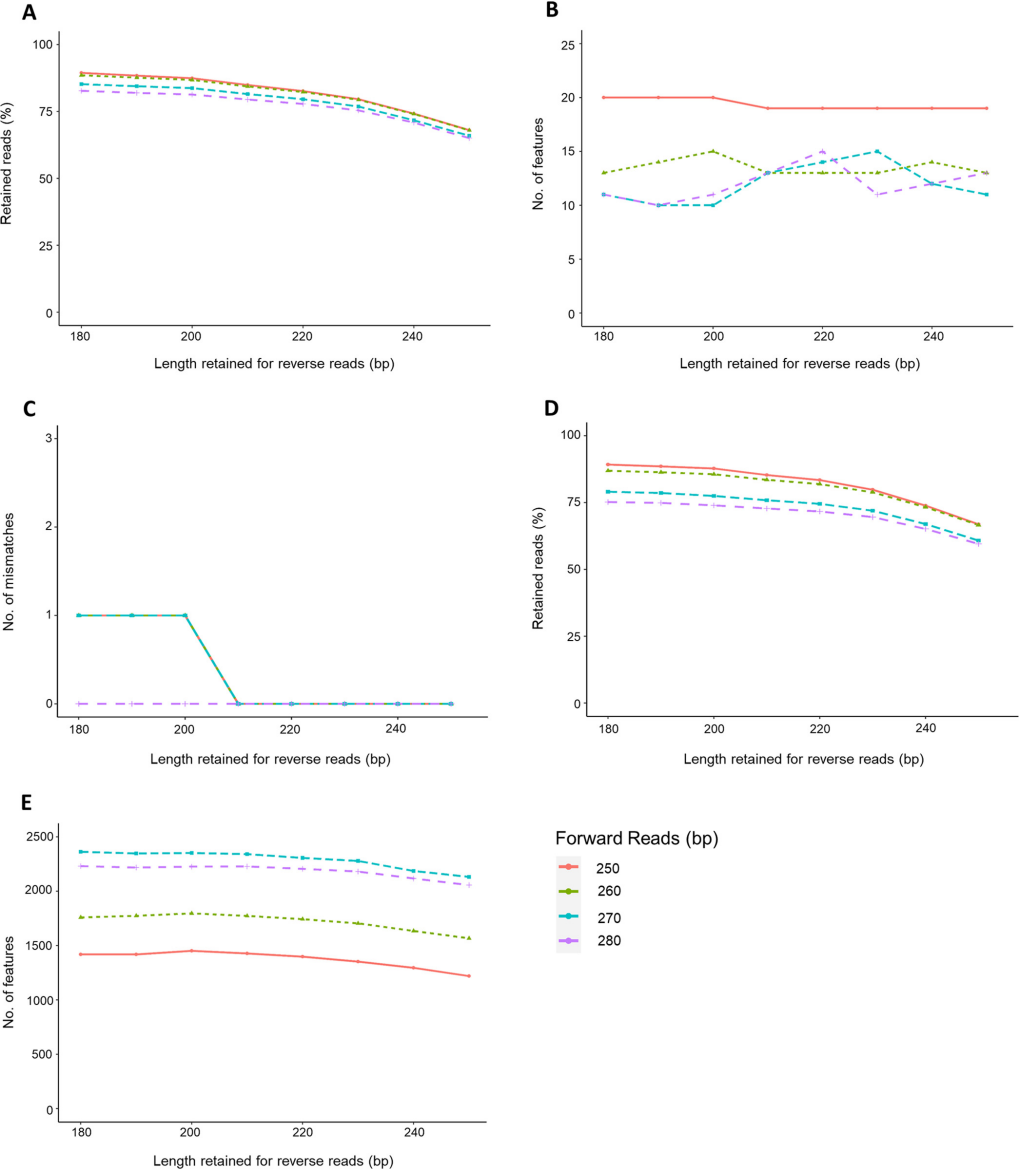
(A and B) The effects of different lengths of forward and reverse reads after truncation on the percentage of sequences retained after denoising (A) and number of features obtained (B) for the ZIEL-I mock community. The numbers of mismatches obtained after local BLAST search against reference sets are shown; these were used in order to test the accuracy of the ASV predictions (C). (D and E) Analysis of human data set on retained reads after denoising and truncation (D) and number of features obtained (E) for each read-length combination.

As clustering methodologies showed a minor influence and the use of different reference databases a more severe impact on taxonomical profiles, we also assessed the potential influence of specific pipeline parameters. As ASVs performed slightly better than zOTUs and OTUs, we focused our comparison on ASVs. Processing steps include removal of primers and adapters, trimming of low-quality reads, chimera removal, and merging of paired-end reads. The removal of all primer and adapter sequences is required for ASV production. Incorrect removal or insufficient trimming leads to loss of sequences in the merging and chimera removal steps. Ambiguous nucleotides would, for example, cause a problem, as default merging settings require a minimum overlap length of 20 bp and identical sequences in forward and reverse reads. Still, we expected the truncation step to have the largest impact on the results. In general, truncation is important to reduce the influence of low-quality bases at the end of the sequence reads. The truncated length for forward and reverse reads can be decided based on two factors: quality scores and amplicon length. However, there is a trade-off between read quality and read length for efficient merging. In this study, we performed the truncation step with different combinations of truncated length for forward and reverse reads for the ZIEL-I mock community for the V4 region (primer pair 515F-806R). Different ranges of forward (250 to 280 bp) and reverse (180 to 250 bp) read lengths were selected based on the quality (*q*) score (≥20) and amplicon length. We found that changes in the forward and reverse truncated lengths directly influence the percentages of sequence counts retained after that step (Fig. 6A). For instance, when the forward read length is set to 250 bp and the reverse read length to be 180 bp, 90% of the input reads were retained. The percentage of retained reads gradually decreased from 90% to 68% when increasing the reverse read length. The same trend was observed for forward 260-bp and reverse truncated length combinations (180 to 250 bp). However, using a forward read length of 270 bp or 280 bp combined with a reverse read length between 180 and 250 bp resulted in a lower percentage of retained reads, ranging from 85% to 65%. The lower number and, thus, reduced percentage of retained reads are mostly due to a decreased number of reads passing the filter. Subsequently, only this decreased number of reads was processed during denoising and merging steps (see Table S3).

The association between the percentage of reads retained and the number of ASVs obtained after those processing steps was also evaluated. The slight differences in the retained percentage of reads for different truncated length combinations did not drastically affect the number of features obtained. The total number of ASVs varied from 10 to 20 for different combinations of truncated lengths for the ZIEL-I mock community. Using truncated lengths of 250 bp and 180 bp for forward and reverse reads, respectively, resulted in 20 ASVs, while other length combinations obtained only 10 to 15 ASVs (Fig. 6B).

To check whether the observed differences in detected ASVs (e.g., 10 versus 20) arose from contaminated reads not corresponding to bacteria included in the ZIEL-I mock community, we performed a local BLAST search. We checked the reads produced by different forward and reverse read combinations against the reference sequence and used a cutoff of ≥97% identity, ≥90% coverage, and E value of ≤0.00001. BLAST results of each forward and reverse read combination showed that 91 to 100% of the ASVs were mapped against the reference sequence of the mock community. The highest number of mismatches was found to be 1 (Fig. 6C). Only a very few nonhits, which did not reach the above-mentioned BLAST cutoffs, were obtained. Nevertheless, truncation for each amplicon length should be tested since low-quality bases impair read clustering.

Mock communities will, irrespective of the number of species added, never fully reflect complex microbial communities. Thus, we analyzed whether truncation showed an impact on a complex microbial community similar to that for the mock community used before. To this end, we used the previously analyzed 33 human stool samples as the test set. We found that the percentage of reads retained after truncation showed lower variations than for the mock community. The largest number of reads retained was identified for setting 250 bp and 180 bp for forward and reverse reads, respectively (Fig. 6D). Interestingly, when using 250 bp for the forward read, the percentage of retained reads decreased from 89% to 67% when increasing the reverse read length from 180 to 250 bp. Thus, insufficient removal of low-quality read sections (i.e., wrong bases) inhibits merging. The number of ASVs varied from 1,219 (250 bp forward/250 bp reverse) to 2,363 (for 270 bp/180 bp) for different combinations of truncated lengths (Fig. 6E), which led us to investigate whether different numbers of ASVs affect taxonomic assignments at genus level. Toward this end, we analyzed the number of generated ASVs for 280-bp forward reads in combination with different reverse read lengths. The number of ASVs varied from 2,057 (for 280 bp/250 bp) to 2,231 (for 280 bp/180 bp). The number of different genera (including unknown and unclassified entries) varied from 131 (for 280 bp/250 bp) to 143 (for 280 bp/190 and 200 bp).

### Selection of primer, pipeline, parameters, and complexity of the ecosystem influences taxonomic classification.

Using three different mock communities, we were able to show differences in taxonomic compositions that were due to differences in used primer pairs, reference databases, clustering methods, or specific settings. We determined a set of bacterial taxa which are biased due to primer choice as well as the reference database (Table 3). Of note, we observed that there is a strong association between the correct assignment of taxa and the complexity of the mock community. For example, *Staphylococcus* was included in all three mock communities. This species was well characterized when using the Zymo mock community but poorly represented when using the more complex mock communities ZIEL-I and ZIEL-II (Table S3). Moreover, we evaluated the influence of specific primers and their comparability in a large population-based cohort (*n* = 1,976 subjects). Amplicon sequencing was performed targeting the V1-V2 and the V3-V4 regions of the 16S rRNA gene (1). For the V3-V4 region, the same primer set as in this work was used. However, for V1-V2, the same primer region was used but the forward primer (27F) did not include the degenerated bases Y and M (80). This led, for example, to a complete loss of identification of *Bifidobacterium* but to an identification of *Akkermansia*. These findings strengthen our hypothesis that methodological settings influence the outcome and, thus, the results that are generated out of 16S rRNA gene sequencing data. We would like to highlight the need for transparency to increase reproducibility and comparability.

**TABLE 3:**
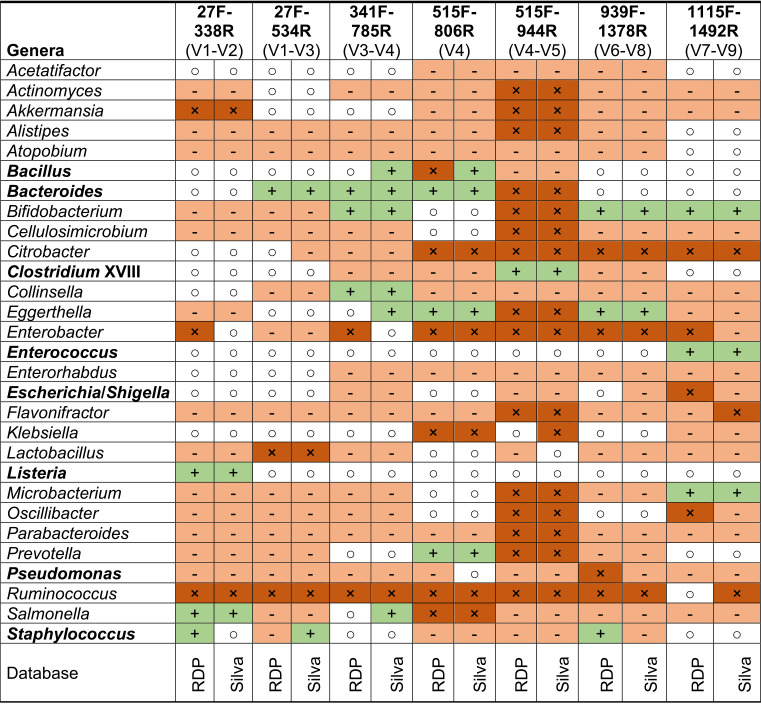
Bacterial taxa at genus level influenced by primer choice and selected reference database^*a*^

a RDP (left column for each V-region) and Silva (right column for each V-region) were used as reference databases. +, <5% difference from reference (shaded green); ○, 5 to 25% difference from the reference (shaded white), −, >25% difference from the reference (shaded light brown); ×, not detected at genus level (shaded dark brown). In bold are bacterial genera present in more than one mock community; therefore, mean values were calculated for these species to estimate their performance.

## DISCUSSION

For short-amplicon 16S rRNA gene sequencing, primers spanning more than one V-region are commonly used, which enhances precision in identifying bacteria compared to a single region. Some of the most frequently used primer pairs enclose V1-V3, V3-V4, and V3-V5, which were used in large population-based cohorts, e.g., the Human Microbiome Project and others (1, 33, 34). Nevertheless, each different primer pair or V-region used will cause bias in the data. In addition, sampling and sample storage, sample processing (including DNA extraction and amplicon generation), sequencing analysis, and data processing introduce further bias. In the last 10 years, many of these factors were studied for a variety of ecosystems, e.g., the human gut (31, 40, 59, 68, 74, 81–84), oral and skin microbiomes (64, 85, 86), food-related ecosystems (87, 88), and environmental microbiomes such as water, marine environments, and sludge (16, 69, 72, 89–91). Nevertheless, the combination of different bias-causing factors was rarely studied. In this study, we analyzed the effects of choice of primer, reference databases, clustering method, and specific pipeline settings in combination on human stool samples and mock communities with increasing complexity using recent approaches. We wanted to highlight the contribution of each these factors to the precision of taxonomic assignment, providing the scientific community with up-to-date guidelines for experimental design and data analysis. Anticipating conclusions, each experimental setting (e.g., cohort and environment) needs to be tested up front for best performance using different experimental settings and strategies.

First, the effect of different primer pairs on the corresponding microbial profile was evaluated. Irrespective of the reference database, the primer pair 341F-785R (V3-V4) slightly outperformed the other combinations and is, therefore, a justified choice for human gut samples. This is also in accordance with Thijs et al. (71), who suggested the primer pair 341F-785R to be a good match for soil and plant-associated bacterial microbiome studies, and Rausch et al. (92), who recommended the use of the V3-V4 region over V1-V2. The sequences produced by using the primer pair 515F-944R (V4-V5) performed well when analyzing the microbiota profile of the Zymo mock community but showed poor performance on the more complex ZIEL-I and ZIEL-II mock communities, suggesting that the primer combination may not be suitable for complex microbial ecosystems at all. This highlights also the importance of including mock communities in routinely performed 16S rRNA gene analysis, as a theoretical sequence analysis by Yang et al. (14) suggested the V4-V5 region to be a good match based on its robustness in representing the full-length 16S rRNA sequences and, therefore, theoretically seemed to be a good primer pair. However, it did not perform well when real samples were used.

Obviously, mock communities do not fully reflect the complexity of a microbial community as it is seen in, e.g., human stool samples. Therefore, we included 33 human fecal samples in our analysis as well. Here, phylum-level classification is robust across the use of different primer pairs targeting different V-regions for *Bacteroidetes* (except 515F-944R), *Proteobacteria*, and *Firmicutes*. In contrast, the detection of *Actinobacteria*, *Tenericutes*, *Lentisphaerae*, and *Verrucomicrobia* varied across the use of different primer pairs, highlighting that the choice of primer should be considered carefully. Intraindividual comparison at genus level showed a high degree of variability across the different targeted regions. This was due to many unknown or unclassified taxa at genus level as well as a generally large number of different taxa. This highlights the need for ecosystem-specific reference databases (93, 94) and new bioinformatic tools that can integrate data across V-regions by taking into account region-specific bias. Here, we notice a need for large-scale studies covering multiple V-regions, which would allow for training taxonomic classifiers that can dynamically account for any region-specific bias. This would possibly be obsolete by sequencing the full-length 16S rRNA gene, although sequencing would still be influenced by the primer choice, i.e., 27F and 1492R, for nearly full-length sequencing. Full-length 16S rRNA gene sequencing is possible by using third-generation sequencing strategies (24, 26, 27) or by the generation of short reads that are later *de novo* assembled to a synthetic full-length sequence (95). Those methods seemingly offer taxonomic identification down to species or even strain level (27). Both approaches are not yet well established for high-throughput sequencing and are not cost-efficient, reproducible, or easy in handling and thus need further investigation to be competitive. Further, long-read sequencing still suffers from comparably high error rates (29, 96).

It is known that the use of different bioinformatic pipelines can have an impact on the determined microbiota composition (40, 43, 65, 97). However, the influence of reference databases for taxonomic prediction was, to our knowledge, not intensively studied. In this study, we evaluated the performance of five different databases using three different mock communities. We tested the ability of each database to identify the correct taxonomy and assessed how well the known diversity of the mock samples could be captured by each database. Our finding illustrated that the Silva and RDP databases were the most accurate 16S rRNA gene databases, showing similar performances consistently superior to those of GRD, LTP, and GG in terms of true positives at the genus level. GG failed to classify *Escherichia/Shigella*, *Listeria*, *Acetatifactor*, *Bacillus*, *Clostridium*, and *Pseudomonas*, in line with the results of Park and Won (98), who found GG to be subpar compared to Silva. GG was last updated in 2013, and any usage is highly questionable.

In addition to the above, we found that quality assessment for each particular database could be conducted only when using a variety of V-regions and a sufficient complex mock community. Low-complexity mock communities using common bacteria did not reveal database issues. Thus, low-complexity mock communities might be used as positive controls in existing pipelines for general quality monitoring, but they are not recommended for detecting fundamental issues when setting up a new study, pipeline, or laboratory. Further, concerning other body sites (or environments), specific mock communities of sufficient complexity should be used. Certainly, the addition of ubiquitous bacteria, like the skin commensal Cutibacterium acnes in humans and other such bacteria, should be considered.

A third factor influencing taxonomic assignment is constituted by the denoising and OTU clustering steps of data analysis. To investigate this aspect, we compared classical OTUs generated by ≥97% clustering Qiime1, ASVs generated by DADA2 denoising (48), and zOTUs generated by the USEARCH denoising algorithm (49, 99). The numbers of features identified by these clustering approaches were nearly identical across all three approaches for the tested mock community. ASV clustering performed well in the human data sets despite the increased complexity, supporting the results of previous studies (42, 100), which suggests that ASVs are the current best choice, as they showed the highest accordance with the theoretical composition of the tested mock community. However, zOTUs performed very similarly and are more robust and user-friendly concerning the input.

Specific settings, e.g., the truncation length, influence the number of reads retained for further analysis steps, as we have demonstrated. Selecting a suitable truncation length is of importance, as too-short reads have short or missing overlaps that lead to problems during merging. Conversely, too-long reads can be difficult to merge, as they show lower sequence quality. The varying number of detected ASVs for different truncation lengths is linked to the trade-off between incorporating reads of lower quality and the sensitivity for detecting low-abundance genera. By systematically reducing the reverse read length, the number of rarely observed sequences increased, as sequencing errors decrease. This highlights an important role for this parameter in the reproducibility of analysis results. To assess this potential bias, we suggest using sufficiently complex mock communities of known composition to determine suitable truncation lengths. Further, it is important to report this parameter (as well as all others) with respect to reproducibility of analysis results.

**FIG 7:**
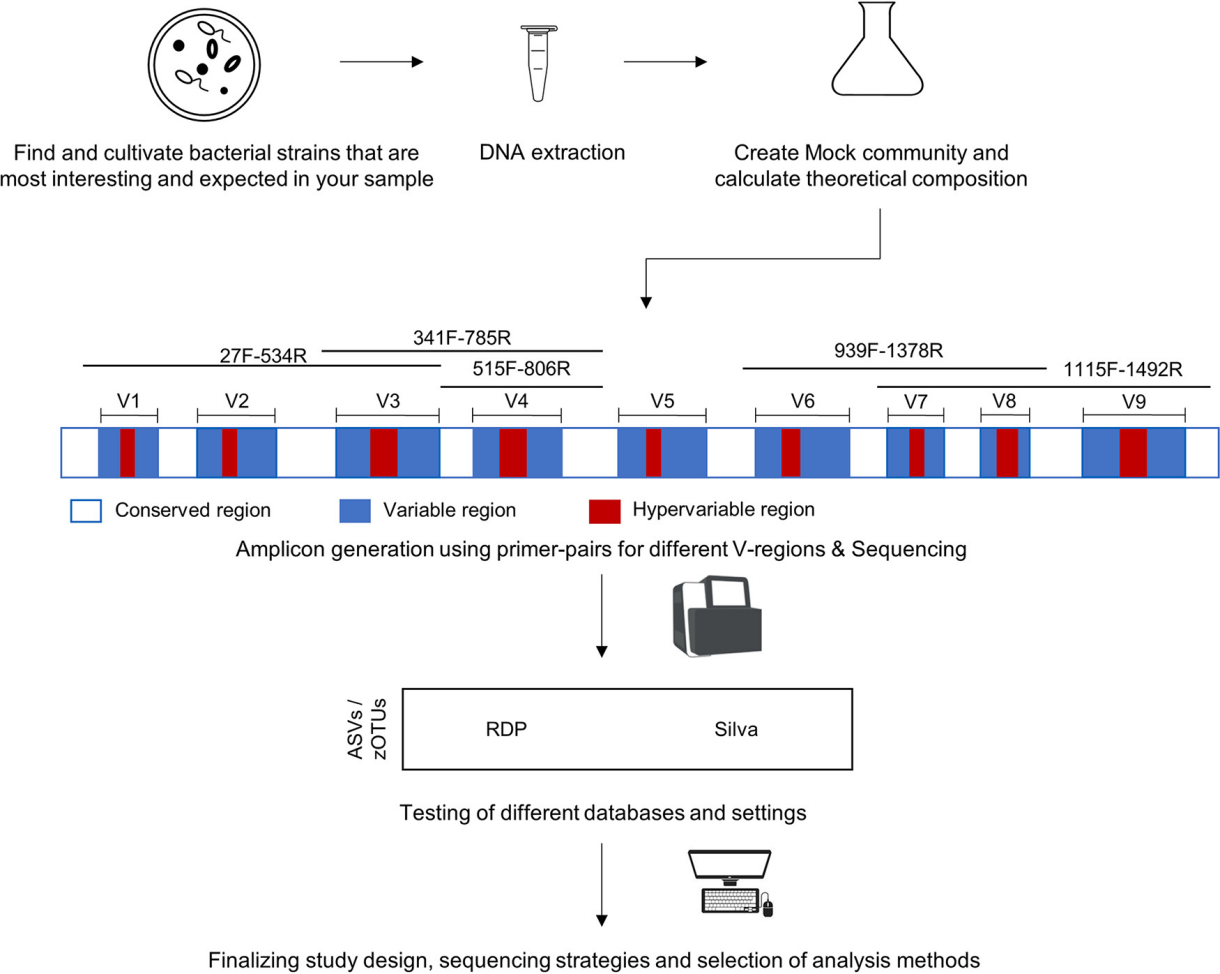
Recommended validation strategy before starting new microbiome studies, especially for uncommon environments. Even existing commonly used parameter combinations might be reevaluated. Thus, complex mock communities should be used and sequenced, testing a variety of different primer pairs for best performance within the environment of interest. Despite their being of minor influence, we still recommend using clustering approaches that include denoising steps (e.g., DADA2 generating ASVs) and recommend the seemingly well-curated and up-to-date databases RDP and Silva as references.

In summary, our results across 3 mock communities and 33 human samples suggest using primers for the V3-V4 region, which show good overall performance for human gut samples. As a reference database, we recommend using either Silva or RDP. Even though only minor differences were observed between clustering methods, we currently recommend using ASVs or zOTUs, with negligible difference between the two. Regarding pipeline settings, we suggest that truncated length combinations should be tested for the primer pairs used in each study. For example, we would suggest for V4 reads truncated to 250 bp and 180 bp for forward and reverse, respectively. However, the last settings depend on the amplicon lengths of the V-regions. To guarantee comparable and reliable results, we recommend creating specific (i.e., reflecting the targeted microbial environment) and sufficiently complex mock community to test whether the study design and the analysis pipelines will be suitable for the bacterial community of interest or type of sample desired (Fig. 7).

## MATERIALS AND METHODS

### Preparation of human gut samples.

Stool samples were obtained from healthy volunteers (33 subjects) and collected in stool sample tubes (Sarstedt AG & Co.). Tubes had been prefilled under a clean bench with 8 ml of stabilizing buffer (1,400 ml of Milli-Q water supplemented with 60 ml of 0.5 M EDTA, 37.5 ml of 1 M sodium citrate, and 1.05 kg of ammonium sulfate [pH 5.2] and sterile filtered using a 0.2-μm filter). A stainless steel mixing bead of 5.5 mm (MP Biomedicals) was added to facilitate homogenization of the crude stool in the stabilizing fluid. The stool was directly resuspended by shaking and vortexing. All samples were aliquoted (in 600-μl portions) and stored at −80°C until DNA extraction.

### Preparation of mock communities.

A mock community is a defined *in vitro*-created mixture of microbial cells. For validation, three different mock communities were used, (i) the ZymoBIOMICS microbial community DNA standard (Zymo Research; catalog no. D6306) with 8 bacterial species, (ii) a more complex in-house mock community (ZIEL-I) including 13 different bacterial species (Table 4), and (iii) another in-house mock community (ZIEL-II) with even more increased complexity including 19 different bacterial species (Table 5). For the in-house mock communities, common gut-related bacterial species were used. The mock community ZIEL-II included such species, which seemed to be influenced by targeted V-region in preliminary results (data not shown). Bacteria were cultured as described in Table S1 and harvested after 2 to 3 days by centrifugation. Pellets were resuspended in stabilizing buffer and stored at −80°C until further processing. After genomic DNA (gDNA) extraction was performed for each strain separately (see below), strain identities were verified by Sanger sequencing. Afterwards, mock communities were constructed by pooling 12 ng of bacterial gDNA per strain. The theoretical composition was calculated according to the formula described for the Zymo mock community by Zymo Research: 16S rRNA gene copy number = total genomic DNA (g) × unit conversion constant (bp/g)/genome size (bp) × 16S rRNA gene copy number per genome. Genome sizes were determined by the 16S reference database EzBioCloud (101). If the genome size for the species included was not available in the database, the closest relative (based on 16S rRNA gene identity) was used for genome size estimation instead. In cases in which only the genus of the bacterium used in the mock community is known, mean genome sizes including all species listed in the database of the genus were used. The 16S rRNA gene copy number was determined from rrnDB (102, 103) as a reference database, also using the closest relative as a surrogate or using mean values of 16S rRNA gene numbers if specific values were not available. Overall, the three different mock communities were sequenced (see below) in duplicates (ZIEL-I) or triplicates (Zymo and ZIEL-II). For further analyses (see below), we used the mean values of the taxonomic compositions of the replicates (all replicates are shown in Fig. S3 to S5).

**TABLE 4:** Composition of the ZIEL-I mock community^*a*^

Species	Amt of gDNA used (ng)	Genome size (bp)	16S rRNA gene copy no.	Theoretical abundance (%)
Actinomyces bowdenii	12	3,103,770	3	6.3
Enterorhabdus mucosicola	12	3,009,822	2	4.3
Cellulosimicrobium cellulans	12	3,850,000	3	5.1
Bacteroides sartorii	12	5,377,291	7	8.5
*Alistipes* sp.	12	3,734,239	2	3.5
Bacillus subtilis	12	4,215,606	9	14.0
Parabacteroides goldsteinii	12	6,751,539	7	6.8
Flavonifractor plautii	12	4,306,691	2	3.0
Clostridium ramosum	12	3,235,195	7	14.2
Enterococcus hirae	12	2,962,227	6	13.3
Acetatifactor muris	12	6,013,646	5	5.4
Staphylococcus warneri	12	2,860,455	5	11.4
*Pseudomonas* sp.	12	6,342,352	4	4.1

a Genome sizes were determined according to entries in EzBioCloud (101), and 16S rRNA gene copy number was determined according to entries in rrnDB (103).

**TABLE 5:** Composition of the ZIEL-II mock community^*a*^

Species	Amt of gDNA used (ng)	Genome size (bp)	16S rRNA gene copy no.	Theoretical abundance (%)
Prevotella copri	12	3,784,859	4	4.2
Collinsella aerofaciens	12	2,463,631	5	8.0
Atopobium parvulum	12	1,543,805	1	2.6
Eggerthella lenta	12	3,500,501	3	3.4
Bifidobacterium longum	12	2,402,802	3	4.9
Clostridium ramosum	12	3,703,302	9	9.6
Staphylococcus epidermidis	12	2,520,741	5	7.8
Klebsiella pneumoniae	12	5,589,189	8	5.6
Escherichia coli LF82	12	4,881,487	7	5.6
Shigella flexneri	12	4,551,801	7	6.1
Oscillibacter valericigenes	12	4,470,622	3	2.6
Akkermansia muciniphila	12	2,760,363	3	4.3
Ruminococcus gnavus	12	3,415,781	5	5.8
Bacteroides vulgatus	12	5,063,322	7	5.4
Pseudomonas aeruginosa	12	6,612,169	4	2.4
Citrobacter freundii	12	5,300,882	8	5.9
Enterobacter cloacae	12	5,030,416	8	6.3
Listeria welshimeri	12	2,819,373	6	8.4
Microbacterium flavum	12	6,818,507	2	1.2

a Genome sizes were determined according to entries in EzBioCloud (101), and 16S rRNA gene copy number was determined according to entries in rrnDB (103).

10.1128/mSphere.01202-20.3 FIG S3 Comparison of Zymo (A), ZIEL-I (B), and ZIEL-II (C) mock sequenced over different V-regions, processed using different databases as references (GG, GreenGenes; RDP, Ribosomal Database Project; GRD, genomic-based 16S ribosomal RNA database; LTP, The All-Species Living Tree Project). The primer pairs span the following V-regions: 27F-338R, V1-V2; 27F-534R, V1-V3; 341F-785R, V3-V4; 515F-806R, V4; 515F-944R, V4-V5; 939F-1378R, V6-V8; and 1115F-1492R, V7-V9. Download FIG S3, PDF file, 0.09 MB. Copyright © 2021 Abellan-Schneyder et al. 2021 Abellan-Schneyder et al. https://creativecommons.org/licenses/by/4.0/ This content is distributed under the terms of the Creative Commons Attribution 4.0 International license.

10.1128/mSphere.01202-20.4 TABLE S1 Culture conditions of bacterial strains used for the ZIEL mock communities. Download Table S1, PDF file, 0.03 MB. Copyright © 2021 Abellan-Schneyder et al. 2021 Abellan-Schneyder et al. https://creativecommons.org/licenses/by/4.0/ This content is distributed under the terms of the Creative Commons Attribution 4.0 International license.

### Extraction of gDNA.

gDNA was isolated with a modified protocol of Godon et al. (104) as described previously (105). Briefly, either 600 μl of pure bacterial culture or 600 μl of frozen stool samples (i.e., bacteria in stabilizer fluid) was thawed on ice and vortexed. Samples were transferred into a 2-ml bead-beating tube (MP Biomedicals), and 250 μl of 4 M guanidinium thiocyanate and 500 μl of 5% sodium *N*-lauroylsarcosine were added. The mixture was incubated at 70°C for 60 min with shaking (700 rpm). Next, cells were disrupted by bead-beating using a FastPrep24 instrument (MP Biomedicals). Bead-beating was conducted three times for 40 s at 6.5 m/s, with cooling with dry ice. Processed samples were stored on ice. Subsequently, 15 mg of polyvinylpolypyrrolidone was added to each sample, with brief mixing. Samples were centrifuged for 3 min at 15,000 × *g* and 4°C, and the supernatant was transferred into a fresh 2-ml sample tube. To every sample, 5 μl of RNase A (10 mg/ml) was added and samples were incubated for 20 min at 37°C with moderate shaking (700 rpm). DNA was purified using gDNA columns (Macherey-Nagel) following the manufacturer’s instructions. Finally, gDNA was eluted in 100 μl of elution buffer provided in the kit. Concentrations and purity were checked using the NanoDrop system (Thermo Scientific), and samples were stored at 4°C (up to 5 days) or at −20°C thereafter.

### Primer selection and *in silico* testing.

Primers for commonly used V-regions were chosen after a literature survey. *In silico* tests of primer specificity were conducted using Silva TestPrime 1.0 (http://www.arb-silva.de/search/testprime/) using standard settings with zero mismatches.

### Library preparation of different variable regions of the 16S rRNA gene.

For amplification of the variable regions (Fig. S1) and addition of adapter binding sites for sequencing, a 1st-step PCR was performed in a 50-μl total volume. Each reaction mixture contained 24 ng of gDNA, 1× Phusion HF buffer, 0.2 mM deoxynucleoside triphosphates (dNTPs), 0.125 μM each forward and reverse primer, 7.5% dimethyl sulfoxide (DMSO), and 0.25 μl of Phusion HF II DNA polymerase (Thermo Fisher). PCR was performed as follows: 98°C for 40 s, 15 cycles of 98°C for 20 s, the V-region specific annealing temperature (Table 1) for 40 s, and 72°C for 40 s, and a final extension step at 72°C for 2 min.

10.1128/mSphere.01202-20.1 FIG S1 Positions of primers mapped onto the Escherichia coli 16S rRNA gene. Graphic designed using SnapGene software (from GSL Biotech). Download FIG S1, PDF file, 0.05 MB. Copyright © 2021 Abellan-Schneyder et al. 2021 Abellan-Schneyder et al. https://creativecommons.org/licenses/by/4.0/ This content is distributed under the terms of the Creative Commons Attribution 4.0 International license.

Barcodes enabling multiplexing were added in the 2nd-step PCR. For this, a 100-μl PCR mixture was prepared using 10 μl of the 1st-step PCR product, 1× Phusion HF buffer, 0.2 mM dNTPs, 0.125 μM each forward and reverse barcode primer, 0.25% DMSO, and 0.5 μl of Phusion HF II DNA polymerase. PCR conditions were 98°C for 40 s, 10 cycles of 98°C for 20 s, 55°C for 40 s, and 72°C for 40 s, and a final extension step at 72°C for 2 min. Further details and work time estimations are found in the work of Reitmeier et al. (105).

### Library quality check and sequencing.

For validation and quality assurance, 8 μl of the 2nd-step PCR product was loaded onto a 1.5% agarose gel. The remaining 92 μl of the 2nd-step PCR product was purified with AMPure XP beads using a ratio of 1.8 times (i.e., addition of 180 μl of beads to 100 μl of sample). Concentrations of the final PCR products were measured in triplicates using a Qubit (Thermo Fisher). Each sample was adjusted to 0.5 nM, and all samples were pooled and sequenced in paired-end modus for 2 × 300 bp (PE300) using a MiSeq system (Illumina, Inc.) following the manufacturer’s instructions. The final DNA concentration of the library was 12 pM, and 15% (vol/vol) PhiX was added.

### Primer-specific feature classifiers.

User-generated feature classifiers accounting for unique characteristics introduced by sample preparation, sequencing primer, and read length perform generally better than the naive classifiers trained on full-length sequences (106). In order to improve the taxonomic classification, five different databases were used to generate primer-specific feature classifiers, namely, GreenGenes (GG) (51), the Ribosomal Database Project (RDP) (52), Silva (53), the genomic-based 16S rRNA Database (GRD) (54), and The All-Species Living Tree (LTP) database (55). Feature classifiers were built for each V-region or primer pair using the *q2-feature-classifier* (107), which is a naive Bayes taxonomic classifier implemented in Qiime2-2019.10 (47).

### OTU clustering using Qiime1.

We consider Qiime-UCLUST (108) a popular example of an OTU-generating method as well as the recently proposed USEARCH-UNOISE3 (49, 99) (described below). Qiime-UCLUST clusters sequence reads at ≥ 97% sequence identity. UCLUST clustering was performed in Qiime1 as follows. Forward and reverse primer sequences and the low-quality reads (q ≤ 20) of demultiplexed paired-end reads were removed by cutadapt 2.10 (109). The trimmed reads were joined by *multiple_join_paired_ends.py* to create a single fasta file of all samples using *multiple_split_libraries_fastq.py*. OTU abundance tables were generated using the UCLUST clustering method through the script *pick_de_novo_otus.py* script in Qiime1. OTU mapping files along with representative sequences, alignment of sequences, and taxonomic alignment files were generated during the *de novo* clustering steps. The RDP database was used as a reference database for defining OTUs at ≥97% sequence similarity.

### zOTU generation using UNOISE.

USEARCH-UNOISE3 aims to reconstruct exact biological sequences from the samples into zOTUs. Paired-end raw reads were merged using the *fastq_mergepairs* script of USEARCH version 11 (108), and the primer sequences were removed using the *fastx_truncate* script. Merging and primer removal steps were conducted before quality filtering, as primer removal reduces the expected errors and merging before quality filtering improves the base call error estimates captured in the overlapping regions as suggested by the author of USEARCH/UPARSE (110). Processed reads were deduplicated and *de novo* clustered into zOTUs. RDP database (project release 11) was used for taxonomic assignment of the representative zOTU sequences.

### ASV generation using nf-core/ampliseq pipeline.

The three mock communities and human data sets were analyzed using the nfcore/ampliseq nextflow pipeline (111, 112). nfcore/ampliseq is a Qiime2-based end-to-end solution for processing 16S rRNA gene amplicon sequencing data. The quality of raw sequencing reads was assessed by FastQC (113). Primer sequences and bases with low-quality scores were trimmed using cutadapt (109). The DADA2 (48) package wrapped inside the nf-core/ampliseq pipeline was used for denoising and constructing ASVs. Based on the quality profile and amplicon length, truncated lengths for forward (250 to 280 bp) and reverse reads (180 to 260 bp) were used in the DADA2 denoising steps to study the relationships between the truncated lengths and number of ASVs generated.

### Data visualization using Rhea.

Data visualization was performed with the R-based pipeline Rhea (114), a collection of R-scripts for 16S rRNA gene sequencing data analysis. After normalization, alpha-diversity and beta-diversity were determined and visualized. Taxonomic classification was conducted down to genus level.

### Data visualization for human samples.

To determine differences of the microbiota composition by targeting different V-regions, a multivariate analysis was performed using the *vegan* R-package. Therefore, a Bray-Curtis distance between samples was calculated based for relative abundance values on phylum and genus levels and grouped according to targeted V-region. First, two dimensions of the nonmetric MDS (NMDS) plot were visualized by using *ggplot2*, and data points were labeled according to targeted V-region.

### Data availability.

Raw sequencing data are available at the Sequence Read Archive under the accession number PRJNA674596.

10.1128/mSphere.01202-20.7 TABLE S4 Comparison of difference to expected theoretical values of the ZIEL-I mock community. In green, lowest difference from ideal amount in mock. Download Table S4, PDF file, 0.09 MB. Copyright © 2021 Abellan-Schneyder et al. 2021 Abellan-Schneyder et al. https://creativecommons.org/licenses/by/4.0/ This content is distributed under the terms of the Creative Commons Attribution 4.0 International license.

10.1128/mSphere.01202-20.8 TABLE S5 Bacterial taxa at genus level influenced by primer-choice and reference database. +, <5% difference from reference (shaded green); ○, 5 to 25% difference from the reference (shaded white); −, >25% difference from the reference (shaded light brown); ×, not detected at genus level (shaded dark brown). In yellow are bacterial genera present in more than one mock community. The primer pairs span the following V-regions: 27F-338R, V1-V2; 27F-534R, V1-V3; 341F-785R, V3-V4; 515F-806R, V4; 515F-944R, V4-V5; 939F-1378R, V6-V8; and 1115F-1492R, V7-V9. Download Table S5, PDF file, 0.2 MB. Copyright © 2021 Abellan-Schneyder et al. 2021 Abellan-Schneyder et al. https://creativecommons.org/licenses/by/4.0/ This content is distributed under the terms of the Creative Commons Attribution 4.0 International license.
