# Supplementary figures and images for: Primer, Pipelines, Parameters: Issues in 16S rRNA Gene Sequencing
Source: mSphere. 2021 Feb 24;6(1):e01202-20. doi: 10.1128/mSphere.01202-20 (PMC8544895; doi:10.1128/mSphere.01202-20)

**A**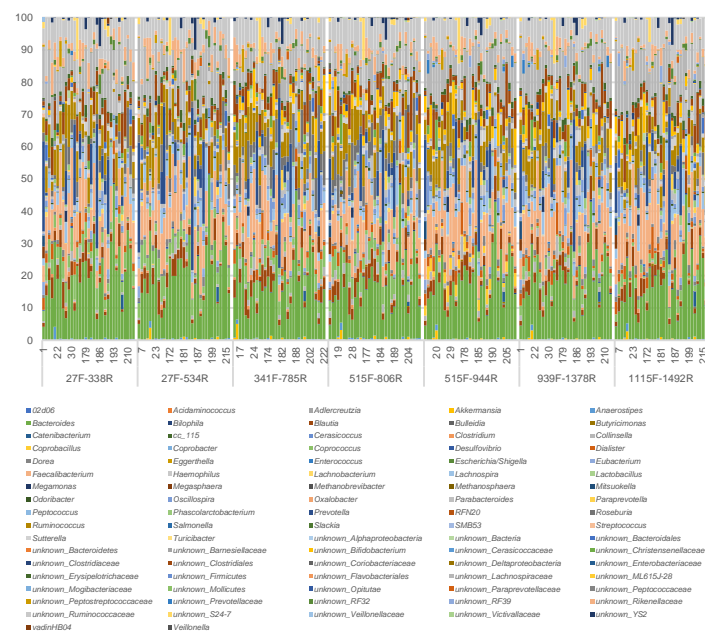**B**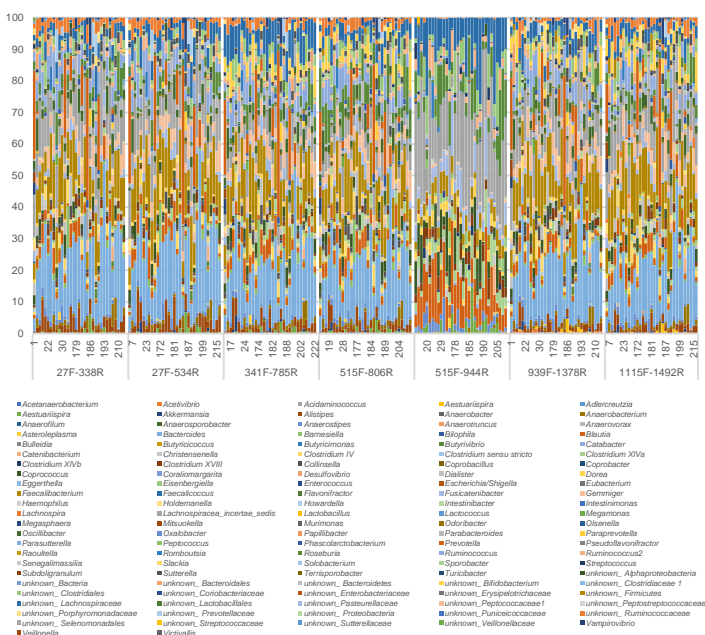**C**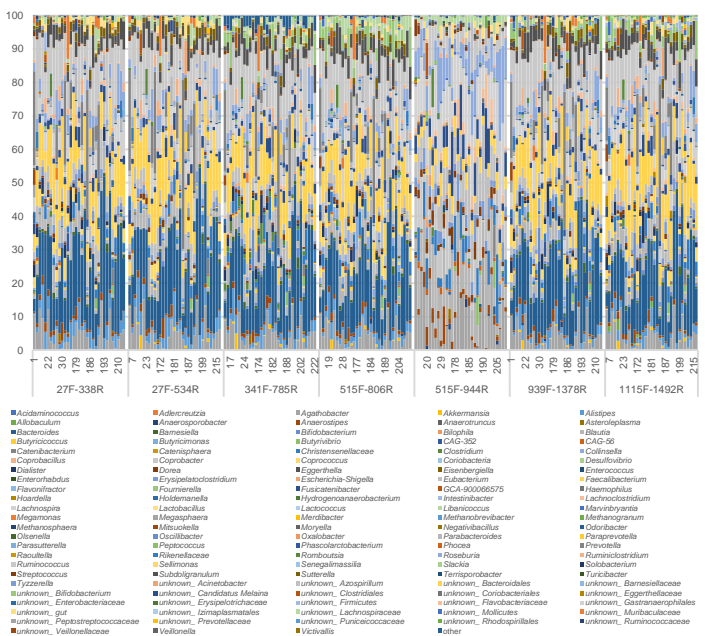**D**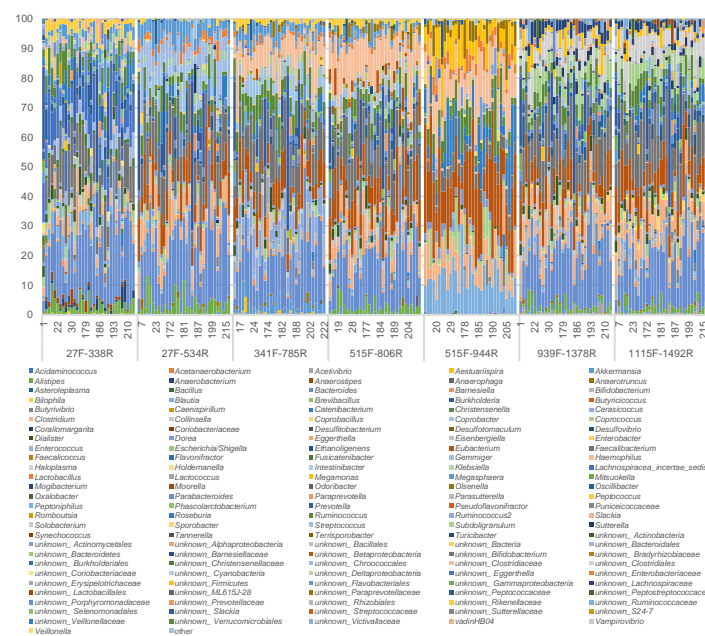**E**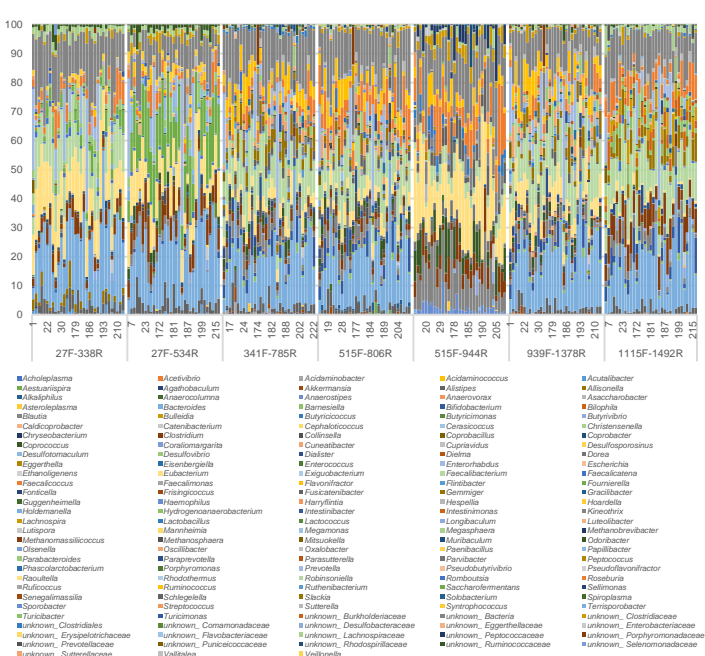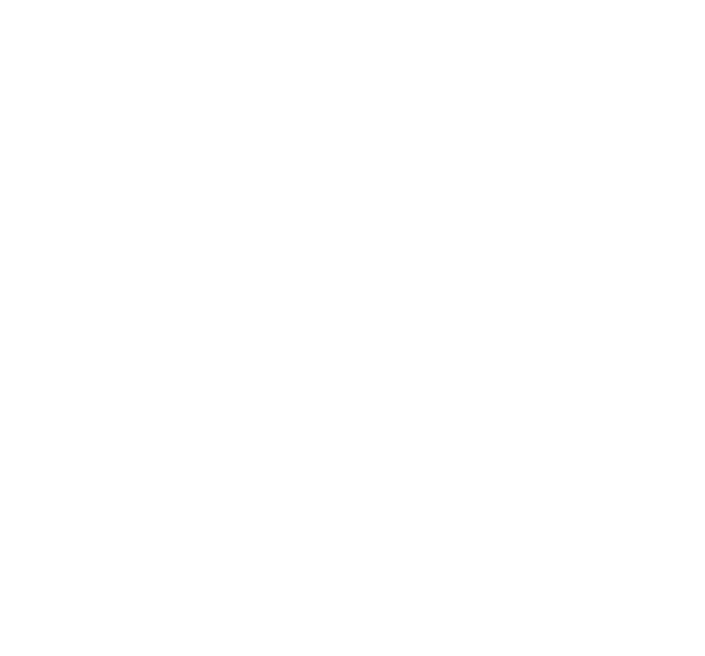

Supplement: FIG S2 [file msphere.01202-20-sf002.pdf]

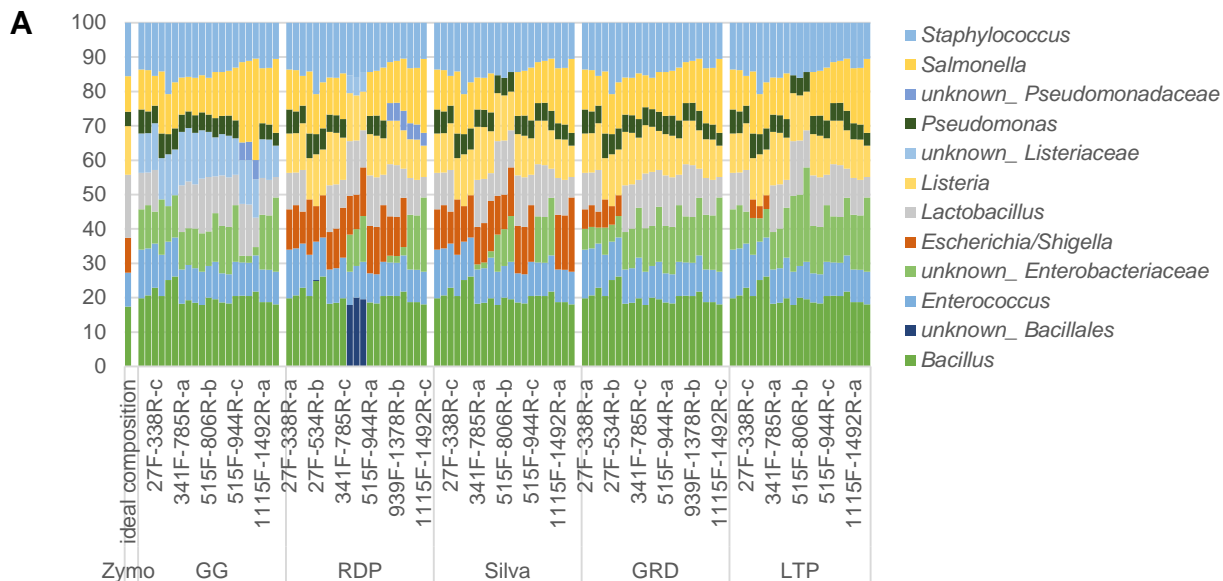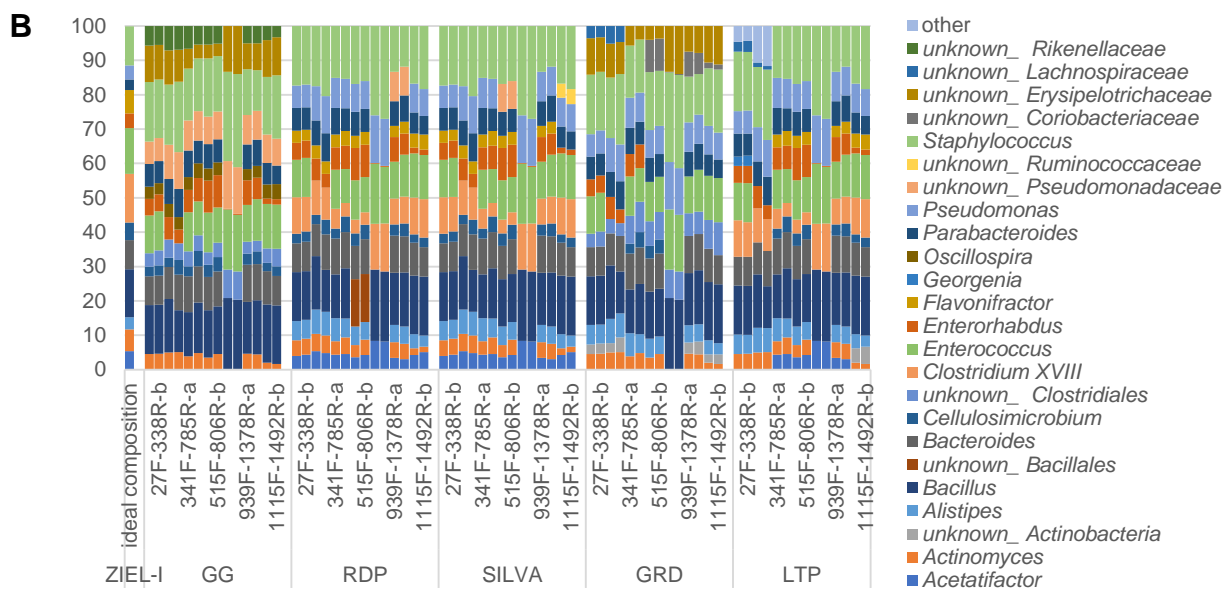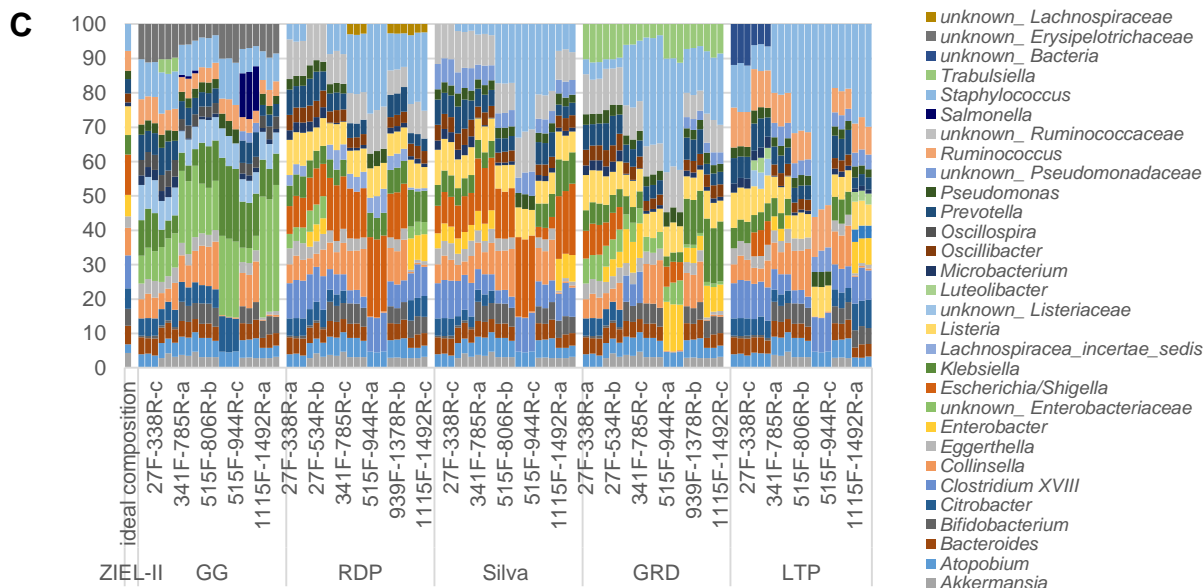

Supplement: FIG S3 [file msphere.01202-20-sf003.pdf]

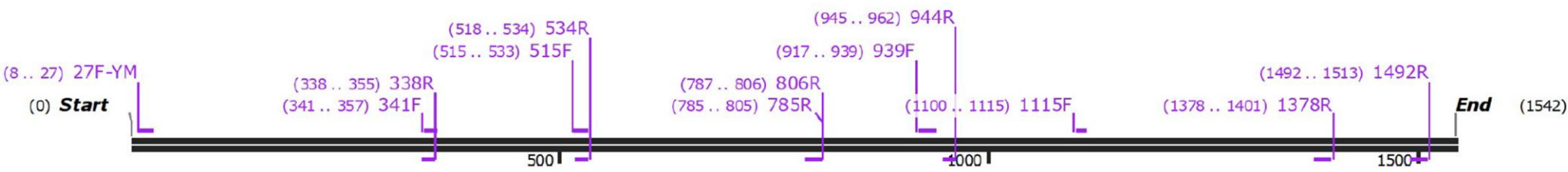

Supplement: FIG S1 [file msphere.01202-20-sf001.pdf]
