# Supplementary material for: Primer, Pipelines, Parameters: Issues in 16S rRNA Gene Sequencing
Source: mSphere. 2021 Feb 24;6(1):e01202-20. doi: 10.1128/mSphere.01202-20 (PMC8544895; doi:10.1128/mSphere.01202-20)
Supplement: TABLE S2 [file msphere.01202-20-st002.pdf]

		Coverage of Kingdom	Coverage of Phyla				
V-Region	Primer	*Bacteria* [%]	*Actinobacteria* [%]	*Bacteroidetes* [%]	*Firmicutes* [%]	*Proteobacteria* [%]	*Verrucomicrobia* [%]
V1-V2	27F-338R	75.9	76	83.6	80.4	81.8	1.2
V1-V3	27F-534R	73.8	69	82.8	78.6	80.7	76.1
V3-V4	341F-785R	82.8	78.1	88.2	83.7	85.5	83.3
V4	515F-806R	82.9	78.2	86.8	84.4	86.1	76.4
V4-V5	515F-944R	48.8	3.7	41.4	60.5	66.6	3.1
V6-V8	939F-1378R	44.6	56.6	50.5	32.2	53	21.2
V7-V9	1115F-1492R	23.1	52.6	0.9	25.7	29.2	6.2
