# Supplementary material for: Primer, Pipelines, Parameters: Issues in 16S rRNA Gene Sequencing
Source: mSphere. 2021 Feb 24;6(1):e01202-20. doi: 10.1128/mSphere.01202-20 (PMC8544895; doi:10.1128/mSphere.01202-20)
Supplement: TABLE S3 [file msphere.01202-20-st003.pdf]

ZIEL-I mock community						
Forward read length (bp)	Reverse read length (bp)	% of input passing filter	% of input de-noised	% of input merged (retained reads)	number of features (ASVs)	number of mismatches (BLAST mismatch)
250	180	90.3	90.0	89.5	20	1
250	190	89.3	89.1	88.5	20	1
250	200	88.3	88.1	87.5	20	1
250	210	85.7	85.4	85.0	19	0
250	220	83.4	83.2	82.7	19	0
250	230	80.3	80.1	79.6	19	0
250	240	74.9	74.6	74.3	19	0
250	250	68.4	68.2	68.1	19	0
260	180	89.2	89.0	88.5	13	1
260	190	88.4	88.2	87.7	14	1
260	200	87.5	87.3	86.8	15	1
260	210	85.1	84.9	84.4	13	0
260	220	82.9	82.7	82.3	13	0
260	230	80.0	79.8	79.4	13	0
260	240	74.7	74.5	74.1	14	0
260	250	68.3	68.1	68.0	13	0
270	180	87.7	87.3	85.2	11	1
270	190	87.1	86.6	84.5	10	1
270	200	86.3	85.9	83.7	10	1
270	210	84.2	83.8	81.5	13	0
270	220	82.2	81.8	79.6	14	0
270	230	79.5	79.0	76.9	15	0
270	240	74.3	73.8	71.7	12	0
270	250	68.1	67.8	66.0	11	0
280	180	85.2	84.8	82.7	11	0
280	190	84.7	84.2	81.9	10	0
280	200	84.1	83.6	81.4	11	0
280	210	82.3	81.9	79.5	13	0
280	220	80.7	80.2	77.8	15	0
280	230	78.3	77.8	75.4	11	0
280	240	73.5	73.1	70.8	12	0
280	250	67.6	67.0	65.1	13	0

Human dataset					
Forward read length (bp)	Reverse read length (bp)	% of input passing filter	% of input de-noised	% of input merged (retained reads)	number of features (ASVs)
250	180	90.6	90.0	89.2	1418
250	190	89.9	89.3	88.6	1418
250	200	89.0	88.4	87.8	1451
250	210	86.6	86.0	85.3	1427
250	220	84.8	84.2	83.4	1398
250	230	81.4	80.8	79.8	1352
250	240	75.6	75.0	73.8	1295
250	250	68.6	68.1	66.9	1219
260	180	89.2	88.2	86.9	1759
260	190	88.6	87.7	86.3	1774
260	200	87.9	86.9	85.6	1796
260	210	85.7	84.8	83.5	1773
260	220	84.1	83.1	81.9	1743
260	230	80.9	80.0	78.8	1704
260	240	75.2	74.4	73.3	1634
260	250	68.4	67.6	66.6	1567
270	180	87.3	84.5	79.0	2363
270	190	86.8	84.1	78.6	2347
270	200	86.2	83.5	77.5	2352
270	210	84.4	81.7	75.9	2341
270	220	82.9	80.2	74.5	2306
270	230	80.0	77.4	72.0	2279
270	240	74.7	72.1	67.0	2186
270	250	68.1	65.6	60.8	2132
280	180	83.9	81.1	75.2	2231
280	190	83.5	80.8	74.9	2218
280	200	83.1	80.4	74.0	2226
280	210	81.7	79.0	72.8	2228
280	220	80.6	77.9	71.7	2206
280	230	78.1	75.5	69.6	2181
280	240	73.4	70.8	65.1	2117
280	250	67.3	64.8	59.5	2057
