# Supplementary material for: Primer, Pipelines, Parameters: Issues in 16S rRNA Gene Sequencing
Source: mSphere. 2021 Feb 24;6(1):e01202-20. doi: 10.1128/mSphere.01202-20 (PMC8544895; doi:10.1128/mSphere.01202-20)
Supplement: TABLE S1 [file msphere.01202-20-st001.pdf]

Mock-community	Name	Aerobe/ anaerobe	Temperature (°C)	Cultivation media
ZIEL-I-mock	*Actinomyces bowdenii*	aerobe	37	TSA/TSB
	Enterorhabdus mucosicola	anaerobe	37	WCA
	Cellulosimicrobium cellulans	anaerobe	37	WCA
	Bacteroides sartorii	anaerobe	37	WCA
	Alistipes sp.	anaerobe	37	WCA
	Bacillus subtilis	aerobe	37	TSA/TSB
	Parabacteroides goldsteinii	anaerobe	37	WCA
	Flavonifractor plautii	anaerobe	37	WCA
	Clostridium ramosum	anaerobe	37	WCA
	Enterococcus hirae	aerobe	37	TSA/TSB
	Acetatifactor muris	anaerobe	37	WCA
	Staphylococcus warneri	aerobe	37	TSA/TSB
	Pseudomonas sp.	aerobe	37	TSA/TSB
ZIEL-II- mock	*Prevotella copri*	anaerobe	37	WCA
	Collinsella aerofaciens	anaerobe	37	WCA
	Atopobium parvulum	anaerobe	37	PYG
	Eggerthella lenta	anaerobe	37	NB / WCA
	Bifidobacterium longum	anaerobe	37	WCA
	Clostridium ramosum	anaerobe	37	WCA
	Staphylococcus aureus	aerobe	37	TSA/TSB
	Klebsiella pneumoniae	aerobe	28	TSA/TSB
	Escherichia coli LF82	anaerobe	37	WCA
	Shigella flexneri	aerobe	37	NB / TSA
	Oscillibacter valericigenes	anaerobe	30	PYG
	Akkermansia muciniphila	anaerobe	37	Schaedler
	Ruminococcus gnavus	anaerobe	37	WCA
	Bacteroides vulgatus	anaerobe	37	WCA
	Pseudomonas aeruginosa	aerobe	37	TSA/TSB
	Citrobacter freundii	aerobe	37	TSA/TSB
	Enterobacter cloacae	aerobe	28	TSA/TSB
	Listeria welshimeri	aerobe	37	TSA/TSB
	Microbacterium flavum	aerobe	28	TSA/TSB
