# Supplementary material for: Primer, Pipelines, Parameters: Issues in 16S rRNA Gene Sequencing
Source: mSphere. 2021 Feb 24;6(1):e01202-20. doi: 10.1128/mSphere.01202-20 (PMC8544895; doi:10.1128/mSphere.01202-20)
Supplement: TABLE S5 [file msphere.01202-20-st005.pdf]

		GG							RDP							Silva							GRD							LTP						
Origin	Genus	27F-338R	27F-534R	341F-785R	515F-806R	515F-944R	939F-1378R	1115F-1492R	27F-338R	27F-534R	341F-785R	515F-806R	515F-944R	939F-1378R	1115F-1492R	27F-338R	27F-534R	341F-785R	515F-806R	515F-944R	939F-1378R	1115F-1492R	27F-338R	27F-534R	341F-785R	515F-806R	515F-944R	939F-1378R	1115F-1492R	27F-338R	27F-534R	341F-785R	515F-806R	515F-944R	939F-1378R	1115F-1492R
ZIEL-I	*Acetatifactor*	×	×	×	×	×	×	×	○	○	○	-	-	-	○	○	○	○	-	-	-	○	×	×	×	×	×	×	×	×	×	○	-	-	-	×
ZIEL-I	*Actinomyces*	-	○	-	-	×	-	-	-	○	-	-	×	-	-	-	○	-	-	×	-	-	-	○	-	-	×	-	-	-	○	-	-	×	-	-
ZIEL-II	*Akkermansia*	×	○	○	-	×	-	-	×	○	○	-	×	-	-	×	○	○	-	×	-	-	×	○	○	-	×	-	-	×	○	○	-	×	-	×
ZIEL-I	*Alistipes*	×	×	×	×	×	×	×	-	-	-	-	×	-	○	-	-	-	-	×	-	○	-	-	-	-	×	-	○	-	-	-	-	×	-	○
ZIEL-II	*Atopobium*	-	-	-	-	-	-	○	-	-	-	-	-	-	○	-	-	-	-	-	-	○	-	-	-	-	-	-	○	-	-	-	-	-	-	○
Zymo	*Bacillus*	-	-	○	○	○	○	○	○	-	○	×	○	○	○	○	-	○	○	○	○	○	○	-	○	○	○	○	○	○	-	○	○	○	○	○
ZIEL-I	*Bacillus*	+	+	+	+	-	○	○	+	+	+	×	-	○	○	+	+	+	+	-	○	○	+	+	+	+	-	○	○	+	+	+	+	-	○	○
ZIEL-II	*Bacteroides*	○	○	○	○	×	○	-	○	○	○	○	×	○	-	○	○	○	○	×	○	-	○	○	○	○	×	○	-	○	○	○	○	×	○	-
ZIEL-I	*Bacteroides*	+	○	○	○	×	-	○	+	○	○	○	×	-	○	+	○	○	○	×	-	○	+	○	○	○	×	-	○	+	○	○	○	×	-	○
ZIEL-II	*Bifidobacterium*	-	-	+	○	×	+	+	-	-	+	○	×	+	+	-	-	+	○	×	+	+	-	-	+	○	×	+	+	-	-	+	○	×	+	+
ZIEL-I	*Cellulosimicrobium*	-	-	-	○	×	-	-	-	-	-	○	×	-	-	-	-	-	○	×	-	-	×	×	-	○	×	×	×	×	×	-	○	×	-	-
ZIEL-II	*Citrobacter*	○	○	+	○	-	×	×	○	○	-	×	×	×	×	○	-	-	×	×	×	×	○	○	×	×	×	×	×	○	○	-	×	×	○	-
ZIEL-II	*Clostridium XVIII*	×	×	×	×	×	×	×	○	+	-	-	○	-	×	○	+	-	-	○	-	○	×	×	○	×	×	×	×	○	+	-	-	○	-	○
ZIEL-I	*Clostridium XVIII*	×	×	×	×	×	×	×	-	-	-	-	+	-	○	-	-	-	-	+	-	○	×	×	×	×	×	×	×	-	-	-	-	+	-	○
ZIEL-II	*Collinsella*	○	-	+	-	-	-	-	○	-	+	-	-	-	-	○	-	+	-	-	-	-	○	-	×	-	-	-	-	○	-	+	-	-	-	-
ZIEL-II	*Eggerthella*	-	○	○	+	×	+	-	-	○	○	+	×	+	-	-	○	+	+	×	+	-	-	○	○	+	×	+	-	-	○	○	+	×	+	-
ZIEL-II	*Enterobacter*	×	×	×	×	×	×	×	×	-	×	×	×	×	×	○	-	○	×	×	×	-	×	-	-	×	-	-	-	×	×	-	×	×	×	-
Zymo	*Enterococcus*	-	○	+	+	○	+	+	-	○	○	+	○	+	+	-	○	○	+	○	+	+	-	○	○	+	○	+	+	-	○	○	+	○	+	+
ZIEL-I	*Enterococcus*	○	×	○	○	-	○	+	○	×	○	○	-	○	+	○	×	○	○	-	○	+	○	×	○	○	-	○	+	○	×	○	○	-	○	+
ZIEL-I	*Enterorhabdus*	○	○	-	-	-	-	-	○	○	-	-	-	-	-	○	○	-	-	-	-	-	○	○	-	×	×	×	×	○	○	-	-	-	-	-
Zymo	*Escherichia/ Shigella*	×	×	×	×	×	×	×	○	-	○	○	-	○	×	○	-	○	○	-	×	-	-	-	×	×	×	×	×	×	-	×	×	×	×	×
ZIEL-II	*Escherichia/ Shigella*	×	×	×	×	×	×	×	-	○	-	○	-	○	×	-	○	-	○	-	-	-	-	-	×	×	-	×	×	×	-	×	×	×	×	×
ZIEL-I	*Flavonifractor*	×	×	×	×	×	×	×	-	-	-	-	×	-	-	-	-	-	-	×	-	×	×	×	×	×	×	×	×	×	×	-	-	×	-	-
ZIEL-II	*Klebsiella*	-	○	○	-	-	-	-	○	○	○	×	○	○	-	○	○	○	×	×	○	-	○	○	○	×	-	-	-	○	○	○	×	×	○	×
Zymo	*Lactobacillus*	-	×	-	○	○	-	-	-	×	-	○	-	-	-	-	×	-	○	○	-	-	-	×	-	○	-	-	-	-	×	-	○	-	-	-
Zymo	*Listeria*	×	×	×	×	×	×	×	○	+	+	○	○	○	○	○	+	+	○	○	○	○	○	+	+	○	○	○	○	○	+	+	○	○	○	○
ZIEL-II	*Listeria*	×	×	×	×	×	×	×	○	○	○	○	+	○	×	○	○	○	○	+	○	○	○	○	○	○	+	○	○	○	○	○	○	+	○	○
ZIEL-II	*Microbacterium*	-	-	-	○	×	-	+	-	-	-	○	×	-	+	-	-	-	○	×	-	+	-	-	-	○	×	-	+	-	-	-	○	×	-	+
ZIEL-II	*Oscillibacter*	×	×	×	×	×	×	×	-	-	-	○	×	○	×	-	-	-	○	×	○	-	-	-	-	○	×	○	-	×	×	-	○	×	○	×
ZIEL-I	*Parabacteroides*	-	-	-	-	×	-	-	-	-	-	-	×	-	-	-	-	-	-	×	-	-	-	-	-	-	×	-	-	-	-	-	-	×	-	-
ZIEL-II	*Prevotella*	-	-	○	+	×	-	○	-	-	○	+	×	-	○	-	-	○	+	×	-	○	-	-	○	+	×	-	○	-	-	○	+	×	-	○
Zymo	*Pseudomonas*	-	-	○	-	-	×	+	-	-	○	×	-	×	×	-	-	○	-	-	-	+	-	-	○	○	-	-	+	-	-	○	○	-	-	+
ZIEL-II	*Pseudomonas*	○	-	○	○	-	×	○	○	-	○	×	-	×	○	○	-	○	○	-	○	○	○	-	○	○	-	○	○	○	-	○	○	-	○	○
ZIEL-I	*Pseudomonas*	×	×	×	×	×	×	×	-	-	-	-	-	×	-	-	-	-	×	-	-	-	-	-	-	-	-	-	-	-	-	-	-	-	-	-
ZIEL-II	*Ruminococcus*	-	○	○	○	+	-	○	×	×	×	×	×	×	○	×	×	×	×	×	×	×	×	×	×	×	×	×	×	-	-	-	-	-	○	-
Zymo	*Salmonella*	+	-	+	○	-	-	-	+	-	○	×	-	-	-	+	-	+	×	-	-	-	+	-	○	+	-	-	-	+	-	○	×	-	-	-
Zymo	*Staphylococcus*	○	○	+	+	○	-	○	○	○	+	+	○	-	○	○	○	+	+	○	-	○	○	○	+	+	○	-	○	○	○	+	+	○	-	○
ZIEL-II	*Staphylococcus*	-	-	○	○	-	○	○	-	×	○	-	-	-	○	×	-	-	-	-	-	+	-	-	-	-	-	-	-	-	○	-	-	-	-	-
ZIEL-I	*Staphylococcus*	-	-	-	-	-	○	-	-	-	-	-	-	○	-	-	-	-	-	-	○	-	-	-	-	-	-	○	-	-	-	-	-	-	○	-
