# Supplementary material for: Primer, Pipelines, Parameters: Issues in 16S rRNA Gene Sequencing
Source: mSphere. 2021 Feb 24;6(1):e01202-20. doi: 10.1128/mSphere.01202-20 (PMC8544895; doi:10.1128/mSphere.01202-20)
Supplement: TABLE S4 [file msphere.01202-20-st004.pdf]

	27F-338R (V1-V2)			27F-534R (V1-V3)			341F-785R (V3-V4)			515F-806R (V4)			515F-944R (V4-V5)			939F-1378R (V6-V8)			1115F-1492R (V7-V9)		
	ASV	zOTU	OTU	ASV	zOTU	OTU	ASV	zOTU	OTU	ASV	zOTU	OTU	ASV	zOTU	OTU	ASV	zOTU	OTU	ASV	zOTU	OTU
Acetatifactor	1.25	1.12	1.45	0.32	1.15	1.39	0.93	1.14	1.22	1.47	1.63	1.76	-2.82	-2.57	-2.05	2.18	2.32	2.19	0.73	0.71	0.83
Actinomyces	1.71	1.78	1.90	1.22	1.72	1.91	1.93	2.33	2.93	2.28	2.48	2.92	6.30	6.30	6.30	1.77	2.05	2.27	4.51	4.77	4.91
Alistipes	-2.07	-1.93	-1.80	-3.52	-2.15	-1.97	-2.56	-2.84	-3.30	-1.78	-1.90	-2.13	3.50	3.50	3.49	-1.55	-1.33	-0.98	-0.19	-0.11	-0.06
Bacillus	-0.22	-0.05	2.46	0.14	0.65	0.75	0.25	0.25	0.38	14.00	0.15	0.12	-6.61	-6.80	-6.80	-1.43	-1.35	-1.18	-3.04	-3.35	-3.25
unknown_ Bacillales	0.00	0.00	-2.33	0.00	0.00	0.00	0.00	0.00	0.00	-13.81	0.00	0.00	0.00	0.00	0.00	0.00	0.00	-1.06	0.00	0.00	-0.02
Bacteroides	0.05	-1.05	-1.33	-1.31	-1.30	-1.18	-1.99	-2.55	-3.16	-1.43	-1.65	-2.13	8.50	8.50	8.47	-2.16	-1.90	-1.58	-0.59	0.01	0.14
Cellulosimicrobium	2.23	2.21	2.25	1.63	1.91	2.04	1.43	5.10	1.64	1.22	1.32	1.44	5.10	5.10	5.10	1.62	5.10	1.89	2.46	5.10	3.01
Clostridium XVIII	3.84	4.47	4.30	4.58	5.99	5.58	9.35	9.51	9.52	10.35	10.26	10.12	0.54	1.00	0.63	6.53	6.77	6.57	3.28	3.65	3.72
Enterococcus	2.16	2.25	2.22	13.30	3.79	3.80	2.68	2.85	2.73	2.45	2.30	2.10	-3.68	13.30	-3.95	1.84	1.60	1.18	0.43	-0.25	-0.64
Enterorhabdus	-0.63	-0.98	-0.67	-0.93	-1.34	-0.71	-2.37	-0.67	-0.04	-5.07	-4.96	-4.34	4.04	4.05	4.08	-2.39	-2.36	-1.95	2.73	3.08	3.28
Flavonifractor	3.42	3.44	3.37	3.05	3.66	3.20	2.78	2.88	2.94	3.15	3.21	3.15	6.80	6.76	6.76	3.46	3.70	3.90	2.52	3.20	3.55
Parabacteroides	-3.55	-3.20	-3.38	-4.74	-3.13	-3.13	-4.17	-4.47	-5.04	-3.86	-4.02	-4.44	3.00	3.00	2.99	-4.41	-5.81	-5.41	-2.80	-3.89	-4.12
Pseudomonas	-2.50	-2.48	-2.23	-6.23	-3.59	-3.70	-4.51	-3.68	-3.41	-3.97	-3.65	-3.48	-9.62	-8.60	-8.01	4.10	-4.09	-4.19	-4.00	-3.85	-3.74
unknown Pseudomonadaceae	0.00	0.00	-0.31	0.00	0.00	0.00	0.00	0.00	0.00	0.00	0.00	0.00	0.00	0.00	0.00	-8.47	0.00	0.00	0.00	0.00	0.00
Staphylococcus	-5.77	-5.60	-6.00	-7.59	-7.40	-8.07	-3.87	-6.30	-6.51	-5.09	-5.25	-5.18	-15.15	-16.40	-17.11	-1.18	-1.45	-1.76	-6.15	-7.05	-7.71
unassigned	0.00	0.00	0.00	0.00	0.00	0.00	0.00	-3.61	0.00	0.00	0.00	0.00	0.00	-17.15	0.00	0.00	-3.31	0.00	0.00	-2.12	0.00
other	0.00	-0.06	0.00	0.00	0.00	0.00	0.00	0.00	0.00	0.00	0.00	0.00	0.00	-0.07	0.00	0.00	0.00	0.00	0.00	-0.02	0.00
